# Nanomaterials Regulate Bacterial Quorum Sensing: Applications, Mechanisms, and Optimization Strategies

**DOI:** 10.1002/advs.202306070

**Published:** 2024-02-13

**Authors:** Chen Hu, Guixin He, Yujun Yang, Ning Wang, Yanli Zhang, Yuan Su, Fujian Zhao, Junrong Wu, Linlin Wang, Yuqing Lin, Longquan Shao

**Affiliations:** ^1^ Stomatological Hospital, School of Stomatology Southern Medical University Guangzhou 510280 China; ^2^ Stomatology Center Shunde Hospital Southern Medical University (The First People's Hospital of Shunde) Foshan 528399 China; ^3^ Hainan General Hospital·Hainan Affiliated Hospital of Hainan medical University Haikou 570311 China; ^4^ Shenzhen Luohu People's Hospital Shenzhen 518000 China

**Keywords:** antibacterial, anti‐virulence therapy, drug resistance, nanomaterials, optimization strategies, quorum sensing

## Abstract

Anti‐virulence therapy that interferes with bacterial communication, known as “quorum sensing (QS)”, is a promising strategy for circumventing bacterial resistance. Using nanomaterials to regulate bacterial QS in anti‐virulence therapy has attracted much attention, which is mainly attributed to unique physicochemical properties and excellent designability of nanomaterials. However, bacterial QS is a dynamic and multistep process, and there are significant differences in the specific regulatory mechanisms and related influencing factors of nanomaterials in different steps of the QS process. An in‐depth understanding of the specific regulatory mechanisms and related influencing factors of nanomaterials in each step can significantly optimize QS regulatory activity and enhance the development of novel nanomaterials with better comprehensive performance. Therefore, this review focuses on the mechanisms by which nanomaterials regulate bacterial QS in the signal supply (including signal synthesis, secretion, and accumulation) and signal transduction cascade (including signal perception and response) processes. Moreover, based on the two key influencing factors (i.e., the nanomaterial itself and the environment), optimization strategies to enhance the QS regulatory activity are comprehensively summarized. Collectively, applying nanomaterials to regulate bacterial QS is a promising strategy for anti‐virulence therapy. This review provides reference and inspiration for further research on the anti‐virulence application of nanomaterials.

## Graphical abstract

This review focuses on the specific mechanisms by which nanomaterials regulate bacterial quorum sensing in the signal supply and signal transduction cascade processes. Furthermore, based on the influencing factors of nanomaterial itself and environment, optimization strategies to enhance the QS regulatory activity are comprehensively summarized. This review provides reference and inspiration for further research on the anti‐virulence application of nanomaterials.

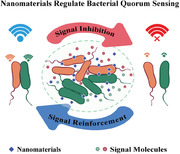

## 1 Introduction

Mankind is facing a post‐antibiotic era, and the World Health Organization estimates that if new antibacterial strategies are not adopted, drug‐resistant bacterial infections may cause ≈10 million deaths per year by 2050.^[^ ^1^ ^]^ Anti‐virulence therapy is a new antibacterial strategy that does not easily induce bacterial resistance. It is not aimed at killing bacteria, but at “disarming” them, thereby preventing them from attacking the host or reducing the severity of bacterial infection, which enhances bacterial clearance by the host immune system.^[^ ^2^ ^]^ Bacterial quorum sensing (QS) regulation is an important anti‐virulence strategy. QS, a major means of bacterial communication, can be used to regulate the expression of hundreds of bacterial genes, thereby affecting behaviors closely related to bacterial virulence, such as biofilm formation, virulence factor production, plasmid conjugation transfer, and bacterial motility.^[^ ^3^ ^]^ Interfering with or blocking QS (quorum quenching [QQ]), can render bacteria deaf‐mute and low‐virulent, unable to resist host immune system “extermination” in a “team warfare” manner.^[^ ^4^ ^]^ Therefore, QQ can serve as a potential alternative treatment strategy or an adjunct to traditional antibiotic therapy, achieving effective anti‐virulence and antibacterial treatment, especially for drug‐resistant bacterial infections.^[^ ^5^ ^]^

Notably, bacterial QS is a cell‐density‐dependent and dynamic multistep process mediated by QS signal molecules. It involves the synthesis, secretion, accumulation, perception, and response of these molecules.^[^ ^6^ ^]^ Each bacterial cell can synthesize and secrete QS signal molecules into the environment as well as monitor changes in the number of bacteria in the population based on the QS signal molecule concentration in the environment. An adequate bacterial abundance triggers an accumulation of QS signal molecules secreted by the bacteria to reach the concentration threshold. At this time, the QS signal molecules can activate the signal perception element and initiate its downstream signal response element to transmit the signal, ultimately regulating the expression of the aforementioned QS‐related genes and coordinating the virulence behavior of the bacterial population.^[^ ^7^ ^]^ The above molecular processes are closely inter‐linked. Therefore, interfering with or blocking any of the steps involved can disrupt the bacterial QS system, greatly weakening bacterial virulence.^[^ ^8^ ^]^

Traditional QS inhibitors (QSIs) are mainly natural products derived from plants and microbes (such as phenolic compounds, terpenoids, alkaloids, and acylase).^[^ ^9^ ^]^ Despite their abundant sources, the use of these traditional QSIs has drawbacks such as low bioavailability (mainly due to poor stability and solubility) and high biological toxicity.^[^ ^10^ ^]^ Due to their unique physicochemical properties and excellent designability, nanomaterials can be used to develop novel types of nanobiomaterials to replace traditional QSIs or achieve efficient delivery of traditional QSIs, which opens up new prospects for QQ‐based anti‐virulence therapy. On the one hand, based on their excellent adsorption, catalytic activity, and optical properties, nanomaterials can directly regulate the key links of QS (including the synthesis, release, accumulation, perception, and response of QS signal molecules), affecting the bacterial group behaviors.^[^ ^11^ ^]^ On the other hand, based on their excellent loading performance and designability, nanocomposites can improve the stability and the targeted delivery efficiency of traditional QSIs components, thereby playing a better role in QS regulation.^[^ ^12^ ^]^ Moreover, nanomaterials can synergize multiple mechanisms through combination strategies to maximize antibacterial effects while reducing the risk of bacterial resistance.^[^ ^13^ ^]^

Because QS is a dynamic multistep process, the specific regulatory mechanism and related influencing factors of nanomaterials in each step are different.^[^ ^14^ ^]^ An in‐depth understanding of the specific regulation mechanism and related influencing factors of nanomaterials in each step is required to optimize the QS regulatory activity of nanomaterials and to develop novel nanomaterials with better comprehensive properties. However, most current reviews were summarized and categorized based on the different chemical components of nanomaterials (such as metal‐based, non‐metallic, inorganic‐based, and organic‐based components) and focused on the synthesis process of nanomaterials, while the content related to their QS regulatory mechanism did not perform a comprehensive and systematic summary and only narratively described the regulatory effects of included different studies one by one; moreover, the existing reviews are limited to the negative regulatory effects of nanomaterials on QS. Therefore, this review first introduces the main application fields of nanomaterials regulating bacterial QS. More importantly, based on each step in the QS process, we comprehensively analyze and summarize the specific mechanisms by which nanomaterials positively and negatively regulate bacterial QS from the signal supply (including signal synthesis, secretion, and accumulation) and signal transduction cascades (including signal perception and response). Moreover, based on the two key factors of “subject‐environment” that affect QS regulatory activity, we propose some more targeted optimization design strategies regarding two factors: nanomaterial‐related and environmental factors. Finally, we highlight the exciting prospects and potential pitfalls of the current research on nanomaterials regulating bacterial QS as well as the solutions and new research ideas. The purpose of this paper is to systematically summarize the specific mechanism of nanomaterials regulating bacterial QS, and to provide a theoretical basis for the development of novel nanomaterials with better QS regulatory activities in the future.

## 2 Applications of Nanomaterials in Regulating Quorum Sensing

**Figure 1:**
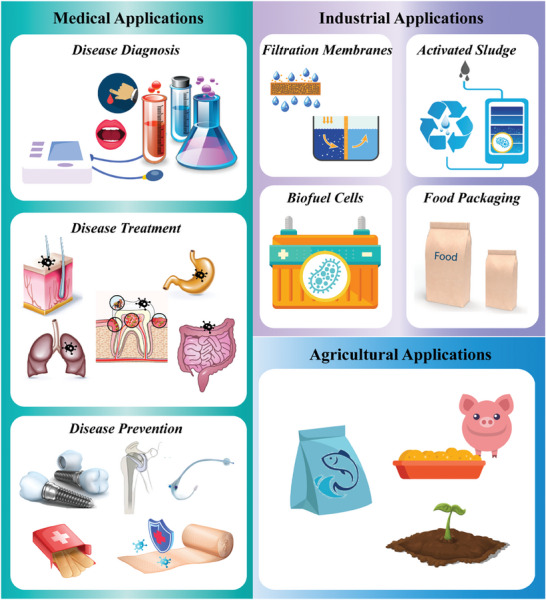
Applications of nanomaterials with QS regulatory activity.

Presently, the use of nanomaterials to regulate bacterial QS has shown broad prospects for application in many fields including medicine, industry, and agriculture (**Figure** **1**) (**Table** **1**).

**Table 1:** Applications of nanomaterials with QS regulatory activity.

Nanomaterials	Bacteria	Advantages	Potential applications	Ref.
CNT‐SPE	*P. aeruginosa*	high sensitivity rapid detection high specificity	electrochemical biosensor rapid diagnosis of infections	[15]
TiO_2_‐Cys NPs	*P. aeruginosa*	easy operation low cost high sensitivity high responsiveness high specificity	optical biosensor diagnosis of infections	[16]
ZnO‐Cys NPs	*P. aeruginosa*	fast response time enhanced sensitivity high specificity	photoluminescence based biosensor early diagnosis of urinary tract infections	[17]
C‐SPE/AuNPs	*P. aeruginosa*	high selectivity and sensitivity rapid and easy identification excellent recycle performance label‐free detection	electrochemical aptasensor identification and characterization of *P. aeruginosa* infections	[18]
PVA/Lap/Au film	*P. aeruginosa*	improved plasmonic sensing ability doubled catalytic rate ease of fabrication	catalysis, sensing, and anti‐microbial surface	[19]
CeO_2_ NCs	*P. aeruginosa, K. pneumoniae, M. mesophilicum, P. gallaeciensis, S. aureus* and *A. tumefaciens*	haloperoxidase mimics low cost good stability high efficiency	antibacterial coating	[20]
MMIP	*A. hydrophila, P. aeruginosa, S. typhimurium, E. coli* and *S. aureus*	real‐time detection capability high specificity excellent reproducibility good stability	electrochemical sensor clinical diagnosis food analysis	[21]
QDs@MIP	*Y. enterocolica, A. hydrophila, P. aeruginosa, S. typhimurium, E. coli* and *S. aureus*	high sensitivity good stability fast response real‐time detection satisfactory recovery	detection of pathogens in food safety and healthcare facilities	[22]
POLE‐capped AgNPs	*C. violacum, P. aeruginosa, K. pneumoniae, E. coli* and *S. aureus*	anti‐metastasis activity anti‐QS and anti‐microbial activity tolerance in vivo	treatment of cancer and secondary bacterial infections	[23]
CAI‐1 NPs	*V. cholerae*	high oral bioavailability great ability to overcome delivery barriers	treatment of cholera	[24]
MPDA‐LUT@CaP NPs	*S. aureus*	stimulus‐responsive release synergistic effects efficient antibacterial capability eliminate biofilm at low temperature effective osseointegration capability	surface coating of Ti implants	[25]
Tob and QSI4 co‐loaded SqNPs	*P. aeruginosa*	significant synergistic effect high potency favorable safety targeted delivery	treatment of pulmonary infections	[26]
SeNPs@HP	*C. violaceum, A. tumefaciens, P. aeruginosa* and *E. coli*	effective intracellular delivery improved anti‐virulence and anti‐biofilm effectiveness enhanced bioavailability	promoting wound healing preventing scars	[27]
nano‐TiO_2_	*E. coli*	quenching AI‐2 signals suppressing biofilm formation no cytotoxicity	anti‐fouling membrane	[28]
AC‐immobilized CNTs	*P. aeruginosa*	high stability effective anti‐biofilm capability enhanced water permeability delay of transmembrane pressure increase	wastewater treatment	[29]
cPANFs of Mag‐EPC	*P. aeruginosa*	high enzyme loading improved enzyme stability reducing biofilm formation higher permeability delay of transmembrane pressure increase	antifouling nanobiocatalyst wastewater treatment	[30]
NER‐AC/Mag‐S‐MPS	*P. aeruginosa*	high stability magnetic separation and sustainability alleviating the biofilm maturation enhanced filtration performance	filtration membrane water reclamation	[31]
NS‐ZnNPs	*C. violaceum, P. aeruginosa, E. coli* and *L. monocytogenes*	broad spectrum no toxicity rapid, cost‐effective, eco‐friendly and safe synthesis process	food packaging	[32]
SLN/Chi/Eu	*P.aeruginosa* and *S. aureus*	optimal stability no cytotoxicity nasal administration sustained and localized release	treatment of pulmonary infections	[33]
Uh‐Au@Nano‐CF	*S. mutans, C. violaceum, E. coli* and *P. aeruginosa*	inhibiting the secretion of virulence factors preventing biofilm formation	prevention of dental infections	[34]
ATNPs	*C. violaceum*	controlled release prolonged residual activity strong anti‐QS activity	food packaging material	[35]
acylase‐Immobilized NF membrane	*P. aeruginosa*	effective anti‐biofilm capability good stability maintained initial flux	wastewater treatment	[36]
r‐AiiA‐MNP	*E. coli*	recyclability reusability great anti‐QS activity	bio‐decontaminating agent	[37]
MNPs	*E. coli*	amplifying QS signaling	facilitating intercellular communication modulating multicellular behaviours	[22]
NCL Nanosuspension	*P. aeruginosa*	pulmonary delivery no acute toxicity low cost	treatment of lung infections	[38]
QSINPs	*P. aeruginosa* and *E. coli*	in‐vivo targeting durable effectiveness excellent anti‐virulence activity	treatment of biofilm‐associated pyelonephritis	[39]
QSI (1) and Tob co‐loaded SqNPs	*P. aeruginosa*	high loading capacity improved biofilm penetration synergistic anti‐biofilm capacity	treatment of pulmonary infections	[40]
Ag‐chitosan nanoparticles	*clinical isolates of S. mutans* and *P. gingivalis*	reducing biofilm formation enhanced biocompatibility corrosion resistance enhanced passivating effect	antibacterial coating	[41]

### 2.1 Medical Applications

In the field of medicine, the main applications of nanomaterials to regulate bacterial QS are in the diagnosis, treatment, and prevention of diseases.

#### 2.1.1 Disease Diagnosis

Using nanomaterials for quantitative analysis of QS signal molecules in body fluids (such as sputum, urine, and plasma) can provide important information for the diagnosis of infections.^[^ ^42^ ^]^ Currently, the nanomaterials used for disease diagnosis based on QS are mostly metal‐based and carbon‐based, and need to satisfy two basic conditions: (1) exhibit selective binding to QS signal molecules in body fluids with high affinity, which enables the concentration of the QS signal to be determined and (2) the ability to amplify the concentration signal and convert it into electrical or optical signals for quantitative detection.^[^ ^43^, ^44^ ^]^ Based on the specific biosensing mechanism, such nanomaterials can be further divided into optical, electrochemical, and bacterial biosensors.

First, certain nanomaterials are widely used in the preparation of optical biosensors due to their special optical properties and ease of functionalization and surface modification.^[^ ^45^ ^]^ Studies have reported the preparation of biosensors (cysteamine‐functionalized ZnO nanoparticles (NPs) and TiO_2_ NPs) based on photoluminescence using microwave‐assisted processes. The capped cysteamine quenches the photoluminescence property of ZnO NPs and TiO_2_ NPs, and QS signal molecules enhance their photoluminescence intensity by increasing the oxygen defect state of the nanomaterial, thereby achieving signal concentration detection.^[^ ^16^, ^17^ ^]^ Notably, the differences in sensing environment and isoelectric points determine the selection of nanomaterials and connecting molecules. The smaller the difference between isoelectric points, the greater the relative response of QS signal detection.^[^ ^16^ ^]^ The inherent luminescence characteristics of quantum dots can also be utilized to detect QS signal molecules. For example, quantum dots coated with molecularly imprinted polymers (QDs@MIP) constitute a new type of specific artificial receptor. When exposed to different concentrations of acyl‐homoserine lactone (AHL), its fluorescence intensity undergoes varying degrees of quenching, making it suitable for signal detection. Compared with the non‐imprinted polymer modified optical biosensors, optical biosensors based on MIPs have superior responsiveness, higher sensitivity, and stronger selectivity.^[^ ^46^ ^]^ Second, nanomaterials can be used for the modification of the electrode surface of electrochemical biosensors. The biosensors modified with nanomaterials demonstrate more stable and repeatable detection performance compared to conventional electrochemical biosensors. This necessitates the nanomaterials to possess good conductivity and strong selectivity for specific QS signal molecules, while also being resistant to interference from other signal molecules, antibiotics, and bioactive compounds in complex environments.^[^ ^15^, ^21^ ^]^ Finally, nanomaterials can be used to prepare biosensors for QS‐active bacterial detection. Unlike optical and electrochemical biosensors, bacterial biosensors require targeted binding to QS‐active bacteria, and the signal requires specific reporting strain perception. Studies have reported the preparation of positively‐charged monodisperse magnetic NPs that can capture negatively charged QS‐active bacteria, aggregate, and couple to the reporting strain under the action of a magnetic field for detecting dispersed pathogenic strains in the commensal microflora.^[^ ^22^ ^]^

It is worth mentioning that the abovementioned detection methods require pathogenic bacteria to reach a certain density before detection. Droplet digital chromogenic assays (DDCA) constitute an effective method for the rapid detection of microorganisms based on the rapid accumulation of chromogenic factors in small droplets.^[^ ^47^ ^]^ In the future, microfluidic chips can be used to integrate the aforementioned nanomaterials with DDCA and develop intelligent biosensors with the ability of low threshold detection.

#### 2.1.2 Disease Treatment

Anti‐virulence therapy using nanomaterials to regulate bacterial QS is a promising infection treatment method. Nanomaterials with QS regulatory activity play an important therapeutic role by weakening the virulence of pathogenic bacteria via three main mechanisms: invasion inhibition, toxin secretion reduction, and biofilm destruction.

First, nanomaterials with QS regulatory activity can weaken the invasion of pathogenic bacteria (such as *Pseudomonas aeruginosa* and *Helicobacter pylori*), preventing them from breaking through the host defense as well as colonizing and disseminating in the body.^[^ ^48^ ^]^ Nanomaterials with QS regulatory activity can prevent pathogenic bacteria from secreting QS‐regulated virulence factors, which contribute to escaping phagocytosis by macrophages and causing tissue damage, thereby enabling bacteria break through the host defense.^[^ ^49^ ^]^ Nanomaterials possessing QS regulatory activity can also weaken the bacterial colonization ability in vivo by downregulating the expression of adhesion factors in pathogenic bacteria. Moreover, the literature suggests that certain bacterial motilities (such as flagella‐mediated swimming and swarming) are closely related to QS regulation, and nanomaterials with QS regulatory activity can interfere with the colonization and dissemination of bacteria by influencing these motilities.^[^ ^50^, ^51^ ^]^ For example, N‐acylhomoserine lactonase (AiiA) stabilized AgNPs can inhibit the autoinducer 2 (AI‐2) QS system of *H. pylori*, downregulating flagellar gene expression (to weaken their motility) and urease production (to weaken their protective ability against gastric acid), thereby controlling *H. pylori* infection in the stomach.^[^ ^52^, ^53^, ^54^, ^55^ ^]^

Second, nanomaterials can alleviate the toxic symptoms of infection by inhibiting the secretion of QS‐regulated virulence factors in pathogenic bacteria.^[^ ^56^ ^]^ Among them, *Vibrio cholerae* is different from most bacteria that activate QS to initiate or maintain virulence. When the level of the QS signal molecule (S)‐3‐hydroxytridecan‐4‐one (CAI‐1) is insufficient, it secretes cholera toxin, causing diarrhea and vomiting.^[^ ^57^ ^]^ Previous study has reported the preparation of CAI‐1 NPs, which have dense polyethylene glycol (PEG) brushes on their surface, making them highly stable and dispersed during transportation from the stomach to the small intestine. Furthermore, they endow nanomaterials with an inert hydrophilic surface to promote drug transport through the mucus layer, activating the QS system of *V.cholerae* to effectively treat cholera.^[^ ^24^ ^]^

**Figure 2:**
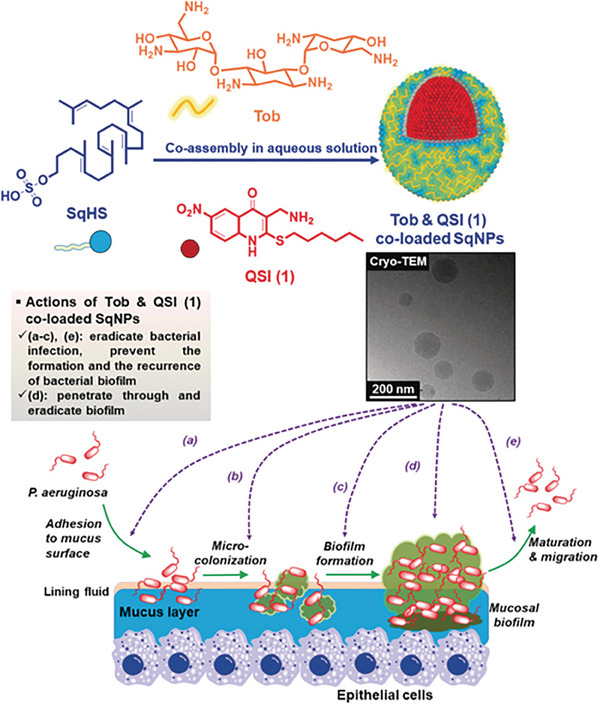
Schematic illustration of the co‐assembly of Tob and QSI (1) co‐loaded SqNPs, their ultrastructure by Cryo‐TEM image, and their proposed actions at all stages of PA respiratory infections. Reproduced under the terms of the Creative Commons Attribution (CC BY) license.^[^ ^40^ ^]^ Copyright 2020, The Authors. Published by Wiley‐VCH Verlag GmbH & Co. KGaA.

Finally, nanomaterials with QS regulatory activity can weaken the defense ability of pathogens by destroying biofilm, thus making infections easier to treat (**Figure** **2**).^[^ ^50^, ^58^ ^]^ First of all, nanomaterials with QS regulatory activity can be used to treat refractory infections related to biofilms, as they can disrupt mature biofilms and assist other antibacterial agents in penetrating the defensive barrier of bacteria, thus exerting synergistic antibacterial effects.^[^ ^59^ ^]^ Researchers have combined photocatalysts and QSIs to prepare a multiple drug‐loaded nanocomposite system for the treatment of peri‐implantitis. Triggered by the weakly acidic biofilm environment, QSIs were released in a controlled manner and selectively acted on biofilms, resulting in a thinner and less dense biofilm structure. Simultaneously applying near‐infrared (NIR) irradiation can completely eliminate the biofilm on the surface of the implant and kill bacteria.^[^ ^25^ ^]^ Second, nanomaterials with QS regulatory activity can be used to treat chronic biofilm‐induced infections, as they can interfere with the dispersion and regeneration process of biofilms by inhibiting the expression of QS‐regulated bacterial surface active molecules and adhesion factors, ultimately preventing repeated and persistent infections.^[^ ^60^ ^]^ Third, nanomaterials with QS regulatory activity can promote the repair of difficult‐to‐heal wounds by clearing mature biofilms or inhibiting biofilm formation, putatively through the following mechanisms: (1) skewing the immune response to prevent biofilms from stimulating the host immune system, inducing inflammatory reactions, and causing damage to surrounding tissues; and (2) protecting cell functional activity and preventing extracellular polysaccharides in the biofilm from inducing cellular senescence as well as interfering with cell reconstruction and tissue repair processes.^[^ ^61^ ^]^ Finally, nanomaterials with QS regulatory activity can also treat oral infectious diseases (such as caries, periodontitis, etc.) by changing the colony structures.^[^ ^62^ ^]^ It has been shown that high concentrations of AI‐2 can accelerate the growth of oral pathogenic bacteria and inhibit the growth of symbiotic bacteria.^[^ ^63^ ^]^ Nanomaterials that can inhibit the synthesis of AI‐2 (such as TiO_2_ NPs) are expected to transform the dominant colonies in oral biofilms from pathogenic bacteria to symbiotic bacteria, thereby controlling the development of caries and periodontal disease.^[^ ^28^ ^]^

The current application of nanomaterials with QS regulatory activity for anti‑virulence therapy is limited to specific digestive and respiratory diseases, probably due to the following reasons: first, nanomaterials can be administered by simple and direct oral or inhalational methods, which ensures sufficient exposure of infection sites to the drug. Second, nanomaterials can overcome the main drug delivery obstacles of digestive system and respiratory system through simple modification and processing. For instance, a two‐step process involving industrially scalable techniques (including high‐pressure homogenization and spray drying) was used to successfully produce niclosamide nanocrystals with smaller particle sizes that can overcome the pulmonary barrier. Reducing the particle size can effectively increase the specific surface area of nanomaterials and enhance their solubility in lung lining fluid. Moreover, the particle size of nanomaterials is lower than the optimal size range for mucociliary recognition and clearance, which can effectively avoid or limit mucociliary clearance and alveolar macrophage phagocytosis, thereby improving drug delivery efficiency.^[^ ^38^, ^64^ ^]^ Additionally, surface modification with PEG can provide a stable and hydrophilic surface for nanomaterials, enabling them to penetrate the mucosal barrier covering the surface of small intestinal crypts directly and regulate pathogenic bacterial QS.^[^ ^24^ ^]^ Third, the pathogenic bacteria are known, and the relevant QS mechanism has been thoroughly studied, which is conducive to designing more targeted nanomaterials.^[^ ^26^ ^]^ In the future, it is necessary to broaden the range of diseases in which nanomaterials can be used for anti‐virulence therapy. For example, creams or gels containing nanomaterials with QS regulatory activity can be developed and used as topical drugs for the treatment of skin and soft tissue infections. Moreover, systemic anti‐QS drugs that inhibit the production of bacterial toxins in the blood can be developed. However, ensuring the in‐vivo absorption, distribution, and effective concentration maintenance of nanomaterial‐containing drugs at a safe dosage remains an important research challenge. Optimizing nanomaterial design, establishing disease research models, and deepening QS mechanistic research may be breakthrough points for solving this problem.^[^ ^40^ ^]^

#### 2.1.3 Disease Prevention

Nanomaterials with QQ activity mainly appear in the form of self‐defensive biomaterials (including implantable and non‐implantable devices) in disease prevention, which exert their effects primarily by inhibiting biofilm formation.^[^ ^65^ ^]^

In implantable devices, nanomaterials mainly play the role of QS regulation in the form of coating, so as to inhibit biofilm formation and prevent the occurrence of biofilm‐related infections.^[^ ^20^ ^]^ Coating the catheter surfaces using nanomaterials with QQ activity (such as Y_2_O_3_:Tb@SiO_2_ nanospheres and PbAg NPs) can effectively inhibit *Proteus mirabilis* QS as well as reduce urease secretion and swarming motility, thereby effectively preventing crystalline biofilm formation in the catheter.^[^ ^66^, ^67^, ^68^ ^]^ Notably, nanocoatings applied in fully implantable devices must not only have QS regulatory properties, but also other characteristics. For example, nanocoatings for cardiovascular implants must have blood compatibility to prevent thrombosis;^[^ ^69^ ^]^ whereas nanocoatings of orthopedic implants should possess bioactivity in order to facilitate vascular and nerve regeneration, calcium and phosphorus enrichment, and osteogenic binding.^[^ ^70^ ^]^ Compared with nanocoatings on the surface of fully implantable devices, those on the surface of partially implantable devices are more susceptible to bacterial contamination in clinical applications due to the need to make contact with the external environment, and thus have higher requirements for QQ activity and antibiofouling ability.^[^ ^71^ ^]^

In non‐implantable devices, nanomaterials mainly play the role of QS regulation in the form of doping and coating, thereby preventing the occurrence of infections.^[^ ^72^, ^73^ ^]^ Among them, doped nanomaterials are mainly used in wound dressings. The doped nanomaterials (such as selenium nanoscaffolds coated with polyphenols of honey [SeNPs@HP]) of wound dressings can prevent wound infection and accelerate wound healing by inhibiting bacterial toxin secretion and biofilm formation; moreover, they can prevent scar formation and improve the quality of wound healing, probably due to the antioxidant, anti‐corrosive, and anti‐inflammatory effects of natural QSI‐based compounds.^[^ ^27^ ^]^ Nanomaterials in the form of coating are mainly used to prevent biofilm‐related infections. Unlike those of implantable devices, the nanocoatings of non‐implantable devices require stronger adhesive forces to resist more complex external stresses.^[^ ^74^ ^]^ Future studies should consider developing nanosurfaces with long‐lasting and stable QS regulatory activity through doping modification, nanoetching technology, or direct nanomaterial‐substrate bonding enhancement. While performing the abovementioned modifications, it is necessary to ensure that the durability, mechanical properties, and biological safety of the material meet the requirements of practical applications.

### 2.2 Industrial Applications

The industrial applications of nanomaterials to regulate bacterial QS mainly include filtration membranes, biological activated sludge, biofuel cells, and food packaging.

#### 2.2.1 Filtration Membranes

Wastewater filtration membranes with QQ activity can prevent a decrease in effluent flux resulting from membrane pore blockage by inhibiting biofilm formation.^[^ ^75^ ^]^ Currently, nanobiocatalytic membranes with QQ activity are mainly obtained by modifying the surface of filter membranes using nanocomposites with QQ enzyme activity.^[^ ^29^ ^]^ Carbon‐based nanomaterials (such as graphite oxide, carbon nanotubes, and carbon fibers), the main materials used for such filter membrane preparation, have the following advantages: first, they have rich and controllable chemical functional groups, which greatly enhances the stability of QSIs in nanocomposites, thereby preventing QQ enzyme inactivation in harsh application environments (such as solid accumulation and shear stress during system operation). Second, a large specific surface area can immobilize a large number of QQ enzymes, which not only successfully solves the problem of limited fixed area on the membrane with high porosity, but also effectively avoids the problem of increased inherent filtration resistance and performance damage when a large amount of QSI is fixed on the membrane surface.^[^ ^29^, ^36^, ^76^, ^77^ ^]^ Besides nanobiocatalytic membranes, QQ enzymes can be fixed on magnetically separable nanomaterials (such as mesoporous silica and carboxylated polyaniline nanofibers) to develop recyclable nanobiological anti‐fouling agents.^[^ ^30^, ^31^ ^]^ However, the production cost of QQ enzymes is high, and it is not easy to maintain enzyme stability. Some nanomaterials (such as photocatalytic and photothermal nanomaterials) can directly quench QS signal molecule activity and inhibit biofilm formation. In the future, such nanomaterials can be used to develop controllable anti‐fouling filtration membranes that do not require the use of natural quenching enzymes.^[^ ^28^, ^37^, ^78^ ^]^

#### 2.2.2 Biological Activated Sludge

Nanomaterials with QS regulatory activity can enhance the biological nitrogen removal processes (including nitrification and denitrification) of biological activated sludge on wastewater, which effectively maintain water cleanliness and prevent water eutrophication.^[^ ^75^, ^79^ ^]^ During nitrification, nanomaterials are mainly used as carriers for the attachment and survival of anaerobic ammonium‐oxidizing bacteria (AnAOB), which increase AnAOB abundance and enhance the density‐dependent nitrification.^[^ ^80^ ^]^ During denitrification, the activation of QS inhibits the denitrification efficiency of bacteria. Nanomaterials can stimulate bacterial denitrification by inhibiting bacterial QS.^[^ ^56^ ^]^ The appropriate dose of nanomaterials with QQ activity should be used to stimulate bacterial denitrification, because high doses of nanomaterials directly disrupt the expression of related genes (including genes coding for proteins involved in nitrogen metabolism, electron transfer, and transport), which are important for bacterial denitrification.^[^ ^81^, ^82^ ^]^ The simultaneous nitrification and denitrification (SND) technology is a new biological denitrification technology involving the use of heterotrophic nitrification‐aerobic denitrification bacteria; this technology has advantages, such as shortened denitrification process, reduced energy expenditure, and simplified system design.^[^ ^83^ ^]^ Future research should focus on the effect of nanomaterials with QS regulatory activity on the nitrogen metabolism of heterotrophic nitrification‐aerobic denitrification bacteria, to further improve the nitrogen removal efficiency of the SND system.

#### 2.2.3 Biofuel Cells

Modifying the electrode surface of microbial fuel cells (MFCs) using nanomaterials with positively QS regulatory activity can effectively promote electroactive biofilm formation on the electrode surface and the electron transport of electroactive microorganisms, thereby improving the electricity production performance of MFCs.^[^ ^84^, ^85^ ^]^ Presently, the nanomaterials used for the surface modification of MFC electrodes mainly include mesoporous nanomaterials, carbon‐based nanomaterials, and metal‐based nanomaterials, whose performance is attributable to the following properties: first, they have a large specific surface area, and thus can provide more space to load more bacteria, thereby initiating bacterial QS and promoting electroactive biofilm formation. Second, they have excellent electron transfer performance, which can reduce charge transfer resistance and improve the charge transfer and current output capabilities of MFC electrodes.^[^ ^86^, ^87^, ^88^, ^89^ ^]^ However, due to the inability of the commonly used nanomaterials mentioned above to overcome the efficiency limitation in the transmembrane electron transfer process, the output power of MFCs has reached the bottleneck.^[^ ^90^ ^]^ One study reported that MFC electrodes can be prepared using “reduced graphene oxide–silver nanoparticle” (rGO/Ag) scaffolds, which could promote the formation of dense biofilms by bacteria while releasing positively‐charged Ag ions (Ag^+^). Bacteria absorb Ag^+^ and reduce them to Ag NPs. Ag NPs, like micro transmission lines, greatly improve the efficiency of transmembrane electron transfer.^[^ ^91^ ^]^

#### 2.2.4 Food Packaging

Incorporating or coating nanomaterials with QQ activity into or on polymers and plastics (such as kaempferol loaded chitosan/TPP NPs, yttrium oxide core/shell nanospheres, and AgCl‐TiO_2_ NPs) can effectively inhibit the biofilm formation and virulence function of food‐spoilage and pathogenic bacteria; thus, these nanomaterials can be used to produce food packaging materials with good food preservation properties.^[^ ^35^, ^66^, ^92^, ^93^ ^]^ Nanomaterials used for food packaging must possess the following characteristics: first, they should be able to withstand harsh food processing conditions and maintain stable QQ activity. Second, they should be able to inhibit the virulence of microorganisms for a long duration and maintain food flavor.^[^ ^35^, ^94^ ^]^ Third, nanomaterial‐improved food packaging materials require other properties, such as flexibility, gas barrier properties, and temperature and humidity stability.^[^ ^95^ ^]^ The development of intelligent nanosensors using nanomaterials with QQ activity, which can exert antibacterial and preservative effects while monitoring the condition of packaged food, is an interesting avenue to explore in future studies; such nanosensors are expected to provide advanced food packaging solutions.^[^ ^32^ ^]^

### 2.3 Agricultural Applications

So far, in the field of agriculture, nanomaterials with QS regulatory activity are mainly used in aquaculture, mainly in the form of animal feed additives. Therefore, besides having anti‐QS activity against pathogenic bacteria (such as *Aeromonas hydrophila*, *Vibrio. parahaemolyticus*, *Vibrio vulnificus*, and *Vibrio harveyi*), the nanomaterials should be safe and non‐toxic to aquatic products.^[^ ^96^ ^]^ Currently, such nanomaterials are mainly biosynthesized using natural QSI compounds as reducing or stabilizing agents, which have good stability, bioavailability, and biological safety.^[^ ^97^ ^]^ Further research has found that biosynthetic nanomaterials with inherent anti‐QS or antibacterial activity may have greater advantages.^[^ ^98^ ^]^ For example, Ag NPs themselves have antibacterial and anti‐QS activity, and research has found that green synthesized Ag NPs from *Gelidiella acerosa* have stronger QQ activity. On the premise of non‐sterilization, it greatly reduces the hydrophobicity, swarming motility, and exopolysaccharide production of bacteria by inhibiting QS, thereby showing excellent non‐bactericidal, anti‐virulence, and anti‐biofilm effects on *V. parahaemolyticus* and *V. vulnificus*. Furthermore, in‐vivo studies have confirmed that the Ag NPs can effectively resist infection and have no toxic effects on the host.^[^ ^99^, ^100^ ^]^ However, the types of nanomaterials with QQ activity that can be applied in aquaculture are currently limited. Some clay minerals can reportedly adsorb QS signal molecules in the environment via their high porosity or catalyze the degradation of QS signal molecules into small inactive fragments via their surface catalytic active sites, which shows an excellent QQ effect on *V. harveyi*. Such minerals are currently used as animal feed additives.^[^ ^101^ ^]^ Future research should consider the use of non‐toxic nanomaterials with similar high porosity or special surface characteristics for the prevention and treatment of bacterial infections in aquaculture.

No current study has evaluated the application of nanomaterials with QS regulatory activity in other fields of agriculture; nevertheless, studies have shown that nanomaterial accumulation in the soil can affect the yield of agricultural crops by regulating various activities of plant growth‐promoting rhizobacteria.^[^ ^102^, ^103^ ^]^ For example, the sub‐inhibitory concentration of Ag NPs can stimulate exopolysaccharide production by the plant growth‐promoting rhizobacterium *Bacillus subtilis*, thereby promoting its effective colonization of plant roots.^[^ ^104^ ^]^ Ag NPs can also stimulate the dissolution of inorganic phosphates by bacteria, thereby increasing the rhizosphere phosphorus availability.^[^ ^104^ ^]^ Therefore, in the future, nanomaterials with QS regulatory activity are expected to be used for agricultural crops; however, prior to such use, it is necessary to evaluate the selective effects of nanomaterials on different rhizosphere microorganisms and their impact on bacterial community composition.

## 3 Mechanisms of Action of Quorum Sensing‐Regulating Nanomaterials

**Figure 3:**
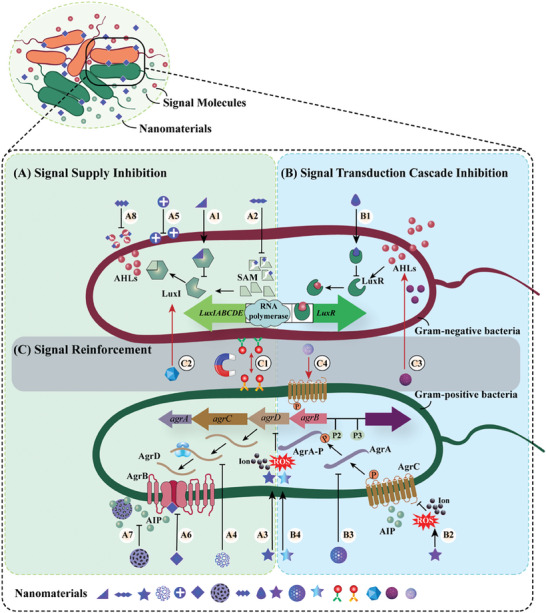
Mechanisms of action of quorum sensing‐regulating nanomaterials. A) represents signal supply inhibition, which includes signal molecule synthesis inhibition (A1, A2, A3, A4), signal molecule secretion inhibition (A5, A6), and signal molecule accumulation inhibition (A7, A8); B) represents signal transduction cascade inhibition, which includes signal perception disruption (B1, B2) and signal response disruption (B3, B4); C) represents positive signal reinforcement, which includes amplifying QS signals (C1), delivering key QS components (C2, C3), and topologically identifying QS receptors (C4). Among them, A1, A2, and A5 are unique regulatory mechanisms of Gram‐negative bacteria, A3, A4, A6, and B3 are unique regulatory mechanisms of Gram‐positive bacteria, and the rest are common mechanisms of both.

Bacteria activate QS mainly by synthesizing, releasing, accumulating, perceiving, and responding to QS signal molecules.^[^ ^105^ ^]^ These QS signaling processes are tightly inter‐linked, and any interference with the molecular processes mentioned above will affect the QS function of bacteria.^[^ ^14^, ^106^ ^]^ Therefore, this section considers the QS signal molecule as the starting point from which to explore the mechanisms involved, and comprehensively summarizes the different mechanisms by which nanomaterials regulate bacterial QS in the above molecular processes (**Figure** **3**). Nanomaterials achieve negative QS regulation mainly through signal supply inhibition (including signal molecule synthesis, release, and accumulation) and signal transduction cascade inhibition (including signal perception and response), to fight against bacteria that employ QS to initiate or maintain virulence. Furthermore, nanomaterials can achieve positive QS regulation by reinforcing QS signals, which are mainly used to enhance specific bacterial group behaviors beneficial to humans.

### 3.1 Signal Supply Inhibition

During the processes of growth and reproduction, bacteria continuously synthesize and secrete QS signal molecules through the catalytic activity of autoinducer synthase or directly through gene transcription and translation. As signal molecules gradually increase in the surrounding environment, when their concentration accumulation reaches the critical threshold, they can be sensed by the corresponding receptors, which initiate the downstream signal response process.^[^ ^107^ ^]^ Nanomaterials mainly inhibit the supply of QS signals by interfering with the three main processes of signal molecule generation, secretion, and accumulation, namely: (1) inhibiting the synthesis of bacterial QS signal molecules to reduce signal molecule generation; (2) inhibiting the transmembrane transport of bacterial QS signal molecules to reduce signal molecule secretion; (3) absorbing or degrading QS signal molecules to inhibit signal molecule accumulation.

#### 3.1.1 Signal Molecule Synthesis Inhibition

QS signal molecule synthesis is the initiating step in the QS process and is associated with various synthases as well as multiple regulated genes, substrates, and precursor molecules.^[^ ^108^, ^109^ ^]^ Nanomaterials inhibit QS signal molecule synthesis mainly by inhibiting enzyme activity and blocking the supply of substrates or precursor molecules, which in turn leads to signal supply inhibition.

Nanomaterials can inhibit enzyme activity by changing enzyme conformation and blocking enzyme active sites.^[^ ^110^ ^]^ Certain metal‐based nanomaterials can directly bind to the surrounding residues of QS‐related enzymes, causing changes in enzyme conformation and decreasing enzyme affinity for substrates or enzyme stability, thereby inhibiting enzyme activity.^[^ ^111^ ^]^ Based on the results of molecular docking, it has been speculated that Ag NPs bind to the catalytic aspartate residue of synthase LasI and histidine residue of RhlI synthase, changing the spatial structure of QS synthase substrate binding site, rendering it unable to bind to substrate molecules, thereby inhibiting enzyme activity and reducing QS signal molecule synthesis.^[^ ^106^ ^]^ Besides changing enzyme conformation, some nanocomposites can also competitively bind to QS‐related enzymes by providing substrate analogues or other precursor molecules and occupying substrate binding sites, resulting in enzyme activity inhibition.^[^ ^111^ ^]^ Some furanone‐related nanocomposites can serve as substrate analogues for S‐ribosylhomocysteinase (LuxS), competitively inhibiting LuxS activity and interfering with AI‐2 synthesis.^[^ ^112^ ^]^

Substrates or precursor molecules (such as S‐adenosyl methionine, precursor peptides) are raw materials for QS signal molecule synthesis.^[^ ^108^ ^]^ Blocking the supply of substrates or precursor molecules is another important mechanism by which nanomaterials inhibit QS signal molecule synthesis.^[^ ^113^ ^]^ First, nanomaterials (such as ZnO NPs and hamamelitannin‐related nanocomposites) can block the supply of precursor molecules by disrupting the expression of precursor peptide genes.^[^ ^58^ ^]^ The possible mechanisms include direct DNA damage, reactive oxygen species (ROS) production causing oxidative damage to DNA, and the release of metal ions that bind to DNA.^[^ ^114^ ^]^ Similarly, fatty acids are the main synthetic precursors of some QS signal molecules (such as AHL and diffusible signal factor),^[^ ^109^ ^]^ and nanomaterials can also block the supply of substrates or precursor molecules by interfering with fatty acid synthesis. The specific mechanisms include: (1) inhibition of key enzymes of the fatty acid synthesis cycle: The type II fatty acid synthesis (FAS II) pathway, which is the necessary pathway for bacteria to synthesize saturated or unsaturated fatty acids, is orchestrated through the sequential cooperation of a series of soluble enzymes encoded by the single gene.^[^ ^115^ ^]^ Triclosan interferes with the key enzyme of the FAS‐II pathway, β‐Enoyl ACP reductase (FabI), inhibiting fatty acid biosynthesis and damaging bacterial cell membranes.^[^ ^116^ ^]^ Lysozyme‐coated Ag NPs can downregulate the key enzyme‐encoding genes associated with the initiation stage and the carbon chain elongation stage of the FAS‐II pathway (including fabA/H/D/G/F/B), thereby interfering with the synthesis of QS signal molecules that use fatty acids as substrates, in a mechanism similar to that of triclosan.^[^ ^117^ ^]^ (2) inhibition of extracellular fatty acid transport: FadL is a pore protein that transports extracellular fatty acids from the outer membrane to the intracellular membranes, where they are activated by the acyl CoA synthase FadD.^[^ ^118^ ^]^ Nanomaterials can prevent extracellular fatty acid transport and inhibit the type II fatty acid biosynthesis pathway by downregulating the expression of *fadL* and *fadD*.^[^ ^117^ ^]^ Interestingly, carbohydrates and branched‐chain amino acids are also synthetic precursors for some QS signal molecules. Some nanomaterials (such as CuO NPs, multi‐walled carbon nanotubes, and Ag nanomaterials) inhibit bacterial QS while also inhibiting bacterial transmembrane glucose transport and enhancing amino acid biosynthesis. Therefore, it is speculated that nanomaterials may also block the supply of raw materials for QS signal molecule synthesis by interfering with amino acid metabolism and glucose metabolism; nevertheless, further research is needed to confirm this speculation.^[^ ^82^, ^119^, ^120^ ^]^

#### 3.1.2 Signal Molecule Secretion Inhibition

Interference with the secretion of QS signal molecules—thereby preventing them from entering the surrounding environment and being recognized by surrounding bacteria—is an important mechanism by which nanomaterials regulate QS.^[^ ^121^ ^]^ Nanomaterials mainly interfere with QS signal molecule secretion by inhibiting the efflux pump system, inducing aggregation effects, and reducing membrane permeability; among these mechanisms, the most extensive and in‐depth research has been performed on efflux pumps.

First, an important physiological function of the efflux pump is the secretion of QS signal molecules.^[^ ^122^ ^]^ Nanomaterials (such as Ag NPs, Au NPs, and TiO_2_ NPs) can interfere with QS signal molecule secretion by inhibiting the efflux pump system, thereby suppressing QS signal molecule supply.^[^ ^123^, ^124^, ^125^ ^]^ The specific mechanisms involve nanomaterials binding with efflux pump proteins, leading to efflux pump blockage; disturbing the transmembrane proton gradient and disrupting the energy supply required for the efflux pump; and suppressing the expression of efflux pump genes. Among them, nanomaterials have a relatively low efflux pump‐blocking efficiency, possibly due to the non‐specific binding between nanomaterials and efflux pump proteins. Targeted nanomaterials can be prepared by combining nanomaterials with anti‐efflux pump monoclonal antibodies or lectins to improve the inhibition efficiency of nanomaterials.^[^ ^126^, ^127^ ^]^

Second, when bacteria display aggregation effects, the extracellular diffusion of QS signal molecules is limited. Nanomaterials can inhibit QS signal molecule secretion via aggregation effect induction by reducing the zeta potential on bacterial surfaces.^[^ ^128^ ^]^ After adsorption onto bacterial surfaces, positively‐charged nanomaterials can neutralize the negative charges on the bacterial surface, resulting in a decrease in the zeta potential of the bacterial surface. When the optimal stoichiometric ratio between nanomaterials and bacteria is reached, the surface charge of the bacteria is fully compensated, i.e., the zeta potential is 0. At this point, bacteria produce aggregation effects and QS signal molecule secretion is limited.^[^ ^129^ ^]^

Finally, the permeability of bacterial cell membranes is crucial for signal molecule secretion through free diffusion. Nanomaterials, such as flavonoid‐based nanocomposites, can rapidly accumulate on bacterial lipid membranes to reduce cell membrane permeability, thereby hindering the outward diffusion of QS signal molecules.^[^ ^128^ ^]^ Apart from pumping out and free diffusion, recent studies have confirmed that vesicular transport is another means of bacterial QS signal molecule secretion. Previous studies have found that nanomaterials can affect vesicular transport in bacteria. In the future, nanomaterials with the function of regulating vesicle transport can be explored to target the secretion of QS signal molecules; however, the specific regulation mechanism involved remains to be further studied.^[^ ^130^, ^131^ ^]^

#### 3.1.3 Signal Molecule Accumulation Inhibition

Nanomaterials can reduce the concentration of available signal molecules in the environment through adsorption and promotion of degradation, thereby preventing them from accumulating to the minimum concentration threshold and initiating downstream signal transduction cascade processes.^[^ ^37^, ^132^ ^]^ Based on different adsorption methods, the adsorption of QS signal molecules by nanomaterials can be divided into physical adsorption and chemical adsorption. Furthermore, based on degradation promotion methods, the degradation‐promoting effect of nanomaterials on QS signal molecules can be divided into enzyme catalysis, photocatalysis, and other methods of natural degradation acceleration.

The different signal adsorption methods are mainly related to the physicochemical properties of the nanomaterials. Nanomaterials with physical adsorption properties (such as graphite oxide and polymer‐based NPs) generally have a large specific surface area and abundant pore structures or specific functional groups that can form hydrophobic bonds with signal molecules.^[^ ^133^ ^]^ Notably, physical adsorption mainly occurs via the intermolecular force between the nanomaterial and signal molecule, and it does not involve electron transfer and chemical bond formation; hence, the adsorption force is relatively weak and reversible.^[^ ^134^ ^]^ Moreover, the adsorption ability of these nanomaterials to signal molecules is limited. Once their maximum adsorption limit is reached, signal molecules undergo desorption, after which the nanomaterials become the source of bacterial signal molecules and instead, promote the QS signal supply.^[^ ^132^ ^]^ The chemical adsorption effect of nanomaterials on signal molecules is mainly related to the organic components modified on their surfaces, among which β‐cyclodextrin is the most studied surface component.^[^ ^135^ ^]^ β‐cyclodextrin has a truncated cone shape with a hydrophobic interior which can serve as the site for complexing various QS signal molecules.^[^ ^136^ ^]^ Compared with physical adsorption, chemical adsorption results in more stable binding to signal molecules and is less prone to desorption. Even if desorption occurs, the structure of the signal molecules has been destroyed and cannot be recognized by bacteria; hence, downstream QS processes cannot be initiated.^[^ ^9^ ^]^

Nanomaterials can promote signal molecule degradation by altering QS signal molecule conformation through various methods such as enzyme catalysis and photocatalysis, among which enzyme catalysis is the most common. First, nanomaterials with enzymatic activity (nanoenzymes) can directly promote QS signal molecule degradation.^[^ ^137^ ^]^ These types of nanomaterials mainly fall into two categories: (1) nanomaterial hybrid enzyme with hydrolase activity through enzyme or enzyme catalytic group modification on nanomaterials: for example, Au NPs degrade AHL by loading the natural enzyme AiiA, resulting in conformational changes in signal molecules, which prevent them from binding to the transcriptional regulator LuxR, ultimately inhibiting QS‐regulated metabolic activity and biofilm formation.^[^ ^138^ ^]^ Molecularly imprinted NPs (MIP NPs) constitute a special type of nanomaterial hybrid enzymes that catalyze QS signal molecule degradation by simulating the shape and position of natural QS‐degradation enzyme active functional groups.^[^ ^139^ ^]^ Earlier work has used the transition state analogue of the γ‐lactone ring hydrolysis as a template to prepare MIP NPs, which can enhance the degradation of N‐hexanoyl‐L‐homoserine lactone (C6‐AHL), a QS signal molecule of Gram‐negative bacteria. Such nanomaterials enhance the stability and durability of modified enzymes or enzyme catalytic groups.^[^ ^140^ ^]^ (2) nanomaterials with inherent enzymatic catalytic properties: These nanomaterials are mainly nanoenzymes with haloperoxidase activity (such as CeO_2_‐X nanorods and V_2_O_5_ NPs), which catalyze the oxidation of halides (Cl^−^, Br^−^, and I^−^) into corresponding hypohalous acid (HOX) intermediates. HOX further catalyzes the halogenation modification of QS signal molecules, leading to inactivation and rapid degradation of QS signal molecules.^[^ ^137^, ^141^ ^]^ Further research has found that this catalytic effect is mainly attributed to the mixed valence state of the nanomaterials, which can generate rapid redox cycling.^[^ ^20^, ^93^ ^]^ Second, nanomaterials (such as TiO_2_ NPs and Y_2_O_3_:Tb@SiO_2_ nanospheres) can also promote the degradation of QS signal molecules through photocatalysis.^[^ ^28^, ^66^ ^]^ When the light energy absorbed by photocatalytic nanomaterials is greater than or equal to their band gap energy, they can be excited to produce free electrons and holes; the oxidative activity of holes and reduction capabilities of electrons are utilized to produce ROS with extremely strong oxidative ability, which plays an important role in promoting the degradation of QS signal molecules. However, the specific mechanism by which ROS promote the degradation of QS signal molecules is still unclear.^[^ ^142^ ^]^ Finally, nanomaterials (such as photothermal nanomaterials) can promote the natural degradation of QS signal molecules by adjusting the environmental parameters around the bacteria (such as temperature and pH).^[^ ^78^ ^]^ The environment is an important factor affecting the natural degradation rate of bacterial QS signal molecules. High temperature and alkaline conditions may induce the opening of the lactone ring and rapid degradation of the QS signal molecule AHL, although this process can be reversed under acidic conditions.^[^ ^143^ ^]^

It should be noted that when using nanomaterials to suppress signal accumulation, the action time of the nanomaterials should be controlled to within an appropriate range. This is because as the interaction time between nanomaterials and bacteria is prolonged, bacteria can enhance the synthesis and secretion of signal molecules through adaptive evolution of QS, thereby resisting the inhibitory signal accumulation effect of nanomaterials.^[^ ^144^ ^]^ The use of nanovaccines to generate antibodies and neutralize QS signal molecules may be a highly promising means of simultaneously inhibiting QS signal accumulation and overcoming adaptive evolution of bacterial QS.^[^ ^9^ ^]^ Nanovaccines not only induce the production of monoclonal antibodies that neutralize QS signals, but also generate immunological memory. Upon subsequent stimulation by the same QS signal molecules, the body can produce a quicker and stronger immune response to neutralize the QS signal molecules, thereby antagonizing the QS signals generated by bacterial reactivity.^[^ ^145^ ^]^

### 3.2 Signal Transduction Cascade Inhibition

When the QS signal molecules in the environment reach a specific concentration threshold, the signal transduction cascade process of bacterial QS is initiated. At this point, QS signal molecules are perceived by bacteria through binding to QS‐related receptors; subsequently, the signal is transmitted through a cascade network, ultimately causing QS‐regulated gene response and expression.^[^ ^146^ ^]^ Nanomaterials can suppress bacterial QS signal transduction cascades by disrupting the perception or response of QS signals.

#### 3.2.1 Signal Perception Disruption

QS signal receptors can be divided into cytosolic receptors and membrane receptors, and nanomaterials can disrupt QS signal perception by interfering with the binding of signal molecules to these two types of receptors.^[^ ^147^ ^]^

**Figure 4:**
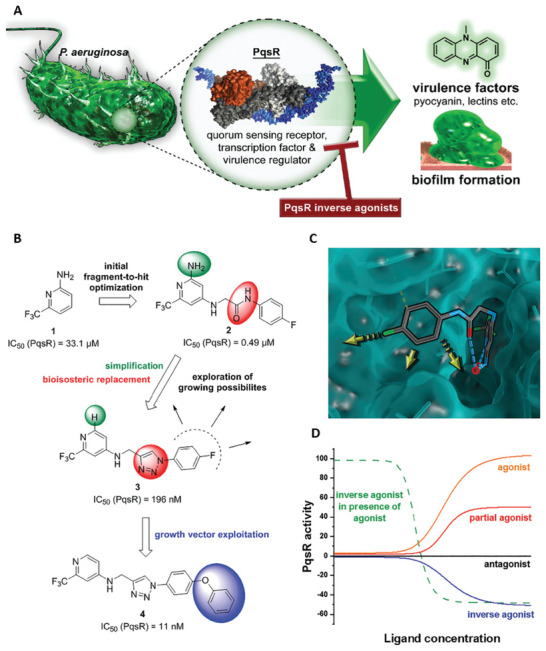
PqsR inverse agonists as pathoblockers and hit‐to‐lead optimization strategy. A) Schematic representation of the mode of action. B) Bioisosteric replacement and structural simplification of hit **2** lead to **3**. The discovery and exploitation of a growth vector‐enabled identification of **4**. C) 3D model of compound **3** in complex with PqsR91‐319 derived from related X‐ray structure (PDB entry 6Q7W). D) Pharmacological profile of various PqsR ligands. Reproduced under the terms of the CC BY license.^[^ ^26^ ^]^ Copyright 2021, The Authors. Advanced Science published by Wiley‐VCH GmbH.

After binding to QS signal molecules, the cytosolic receptor binds to the promoter or RNA polymerase of the target gene in the form of a dimer to regulate the transcription of QS‐regulated genes.^[^ ^108^ ^]^ Nanomaterials mainly inhibit the binding of QS signal molecules to the cytosolic receptor through competitive and noncompetitive means. First, the structural analogues of signal molecules can competitively bind to QS signal receptors. Nanomaterials using signal molecule analogues as capping agents or nanocomposites loaded with analogues can competitively bind to receptors, thereby occupying the binding sites of signal molecules, preventing receptors from binding to these molecules, resulting in the loss of their transcriptional regulation function (**Figure** **4**).^[^ ^26^, ^128^ ^]^ For example, many studies have developed nanomaterials with anti‐QS activity using polyphenols (such as caffeic acid, quercetin, apigenin, kaempferol, chrysin, and luteolin) as capping agents or loaded drugs.^[^ ^111^ ^]^ Further analysis of the structure‐activity relationship has revealed that the aromatic ring conjugated domain of the polyphenols is similar to the homoserine lactone part of the QS signal molecule, which can bind to the signal molecule binding site of the receptor through hydrogen bonding and hydrophobic interactions, thereby occupying this site.^[^ ^27^ ^]^ Due to the lack of binding of real QS signal molecules, the receptor loses its ability to initiate QS‐regulated gene expression. Furthermore, nanomaterials can bind to sites other than the signal molecule binding site of the receptor in a noncompetitive manner and directly modify the receptor or receptor signaling complex, resulting in a decline in its stability and rapid hydrolysis, or making it unable to interact with the promoter or RNA polymerase of the target gene because of conformational changes; these effects result in blockade of QS signal transduction cascades.^[^ ^13^, ^23^ ^]^ For example, flavonoid‐loaded nanocapsules bind to the domain of non‐signal molecular binding sites on the receptor LuxR, resulting in conformational changes in LuxR, thus preventing it from binding to the DNA promoter.^[^ ^128^ ^]^ Besides, nanomaterials themselves can also noncompetitively inhibit the binding of signal molecules to QS receptors.^[^ ^106^ ^]^ Molecular docking analysis suggests that Ag NPs may bind to the leucine residues of LasR and tryptophan and glutamate residues of RhlR, causing the failure of related receptors to bind to the target genes, thereby inhibiting downstream QS gene expression.^[^ ^106^ ^]^ The inhibition of intracellular transport of signal molecules is also an important mechanism for inhibiting their binding to QS receptors.^[^ ^113^ ^]^ It has been found that apolipoprotein B can chelate QS signal molecules in the surrounding environment and prevent signal molecules from being transported into bacteria.^[^ ^148^ ^]^ Similarly, *Escherichia coli* secretes LsrK, which phosphorylates extracellular AI‐2 signals and prevents them from being transported into the cytosol.^[^ ^149^ ^]^ Based on this mechanism, future studies should design nanomaterials that can interfere with the intracellular transport of QS signal molecules to regulate bacterial group behaviors.

Like cytoplasmic receptors, membrane receptors (mainly two‐component systems) also play an important role in QS signal perception. It has been reported that nanomaterials can block QS signal perception by inhibiting membrane receptor binding to signal molecules.^[^ ^150^ ^]^ Metal‐based nanomaterials or their dissolved metal ions adsorbed on the bacterial cell membrane can bind to the amino (‐NH) and carboxyl (‐COOH) groups of the membrane receptor protein with high affinity, resulting in the structural and functional destruction of the membrane receptor.^[^ ^151^ ^]^ For QS‐related membrane receptors with similar groups, nanomaterials may also destroy the receptor structure through a similar mechanism, thereby disrupting the receptor ability to bind to QS signal molecules.

#### 3.2.2 Signal Response Disruption

Inhibition of the expression of genes regulated by QS systems at transcriptional and translational levels is the most important mechanism by which nanomaterials directly disrupt signal response; in this process, nanomaterials directly destroy intracellular biomacromolecules that are vital to target gene expression through physical and biochemical processes.^[^ ^152^ ^]^ It should be noted that nanomaterials can modulate QS by simultaneously regulating one or more of these pathways. The QS system complexity enables different cascades to bypass the suppressed pathway, and the nanomaterials that simultaneously regulate multiple pathways through multiple mechanisms can minimize drug failure caused by the QS system complexity.^[^ ^81^ ^]^

At the transcriptional level, nanomaterials mainly destroy QS‐regulated genes by direct damage, ROS‐mediated oxidative damage, and chelation of released metal ions, thereby inhibiting the transcription process.^[^ ^69^ ^]^ First of all, nanomaterials can be directly inserted between DNA base chains, resulting in gene damage; they can also bind to DNA, thereby triggering DNA condensation, which impedes DNA replication.^[^ ^153^ ^]^ Second, nanomaterials can induce ROS production and the oxidative damage of QS‐regulated genes.^[^ ^81^ ^]^ Glutathione is an important ROS scavenger. Ag NPs can combine with reduced glutathione to weaken bacterial ROS scavenging ability, resulting in ROS accumulation and aggravating oxidative damage to genes.^[^ ^81^ ^]^ Finally, metal‐based nanomaterials entering the cell can slowly dissolve and release metal ions, which can directly bind to the phosphorus in the gene, thereby breaking the DNA chain due to phosphodiester bond destruction.^[^ ^154^ ^]^ In addition to the DNA template, QS‐regulated gene transcription requires nucleotides synthesized by folic acid metabolism as raw materials for mRNA synthesis.^[^ ^155^ ^]^ It has been shown that certain nanomaterials can interfere with folate metabolism and nucleic acid biosynthesis by disrupting dihydrofolate reductase and dihydropteroate synthase. Such nanomaterials may interfere with bacterial nucleotide biosynthesis, hindering QS‐regulated gene transcription.^[^ ^156^ ^]^ Moreover, the initiation of QS‐regulated gene transcription requires RNA polymerase activation. Whether nanomaterials can inhibit the transcription of these genes by destroying RNA polymerase activity has not been reported.^[^ ^108^ ^]^ The σ factor can reversibly bind to the active catalytic site of RNA polymerase core enzyme, and only the combined RNA polymerase can recognize the promoter and activate the initial transcription of genes.^[^ ^157^ ^]^ The σ factor may be an important target for nanomaterials to regulate RNA polymerase activity.

At the translation level, the specific mechanisms by which nanomaterials inhibit the expression of QS‐regulated genes are as follows: (1) modifying the mRNA sequence to block the binding of the mRNA translation start site to the ribosome;^[^ ^56^ ^]^ (2) preventing rRNA from binding to ribosome subunits to interfere with ribosome assembly;^[^ ^158^ ^]^ (3) producing intracellular toxicity to denature ribosome;^[^ ^159^ ^]^ (4) inhibiting the expression of ribosome‐encoding genes.^[^ ^160^ ^]^ Notably, small regulatory RNAs (sRNAs) can inhibit or activate protein translation processes by binding to mRNA, thereby participating in the QS regulatory pathway and enabling bacteria to perceive population density.^[^ ^161^ ^]^ Moreover, a study found that bacterial sRNA expression levels changed under the action of nanomaterials (such as Ag NPs and titanium dioxide NPs).^[^ ^162^ ^]^ Nanomaterials are likely to regulate bacterial response to QS signals at the translation level through sRNA, but further research is needed to confirm this hypothesis.

Aside from inhibiting QS‐regulated gene expression, blocking intracellular signal transmission is an effective means of disrupting bacterial response to QS signals. Bacteria need to transfer phosphate groups and energy through multistep phosphorelay to achieve intracellular QS signal transmission.^[^ ^163^ ^]^ It has been shown that phosphatase‐like nanozymes have dephosphorylation modification functions, and such nanomaterials may block the intracellular QS signal transmission by removing the phosphate groups of QS responsive elements, thereby disrupting bacterial QS signal response.^[^ ^154^ ^]^

### 3.3 Positive Signal Reinforcement

Besides the negative regulation of bacterial QS, nanomaterials can also positively regulate bacterial QS.^[^ ^24^, ^85^ ^]^ The positive regulation of bacterial QS with nanomaterials offers several advantages as follow: (1) promoting the formation of beneficial biofilm: positive regulation of bacterial QS can effectively promote the formation of electroactive biofilms, which is of great significance to optimize the conversion performance of electroactive bacteria and improve the production capacity of biofuel cells. Furthermore, positive regulation of intestinal probiotic QS (such as AI‐2/LuxS QS system of *Lactobacillus rhamnosus* GG) can promote the formation of probiotic biofilms, thus strengthening the immune functions of the intestinal barrier and promoting intestinal health.^[^ ^84^, ^164^ ^]^ (2) optimizing the bacterial population structure: positively regulating the QS of targeted microbiota (such as AnAOB and starter bacteria) can regulate the bacterial population structure and increase the abundance of the targeted microbiota, which has great application potential in the field of biodegradation and microbial fermentation.^[^ ^80^, ^165^ ^]^ (3) visualization of bacterial virulence activities: by amplifying and transforming the QS signal of pathogenic bacteria in clinical samples or food into visualized signals, the virulence activities of pathogenic bacteria can be reflected dynamically; thus, the sensitivity of bacterial detection techniques can be greatly improved.^[^ ^21^ ^]^ (4) promoting the synthesis of target bioactive agents: some target bioactive agents are bacterial secondary metabolites regulated by QS system, and the production efficiency of these bioactive agents can be effectively improved by positively regulating bacterial QS.^[^ ^166^ ^]^ (5) promoting bacterial non‐virulent behaviors: activation of QS systems can promote the transformation of certain bacteria (such as *V. cholerae*) from virulent to non‐virulent states, thus playing a role in disease treatment.^[^ ^24^ ^]^ Nanomaterials mainly regulate bacterial QS through strategies such as amplifying QS signals, delivering key QS components, and topologically identifying QS signal receptors.

First, nanomaterials can amplify QS signals at lower bacterial densities by directly altering the spatial distribution of bacteria, thereby reducing the distance between bacteria.^[^ ^22^ ^]^ This strategy, which can activate bacterial QS without expensive and complex operations (such as the exogenous addition of QS inducers), has enormous potential application value. Studies have reported advancements in the development of positively‐charged magnetic NPs (MNPs) which capture negatively charged QS‐active bacteria via electrostatic interactions; these bacteria are thus aggregated via a magnetic field. Due to the shortened distance between bacteria, the local concentration of QS signal molecules secreted by captured bacteria increases, and the QS signal is amplified.^[^ ^22^ ^]^ However, this QS signal amplification strategy based on electrostatic interactions has difficult problems in selectively amplifying specific QS signals and low amplification efficiency due to the inability of nanomaterials to specifically bind to target bacteria. The QS signal amplification strategy based on specific antibody‐antigen binding can effectively solve these problems. Antibody‐coated MNPs have been developed that can specifically capture and concentrate signal‐sending bacteria and signal‐receiving bacteria through antigen‐antibody interactions, thereby improving the bioavailability of QS signal molecules from sender to receiver and specifically amplifying QS signals.^[^ ^167^ ^]^

**Figure 5:**
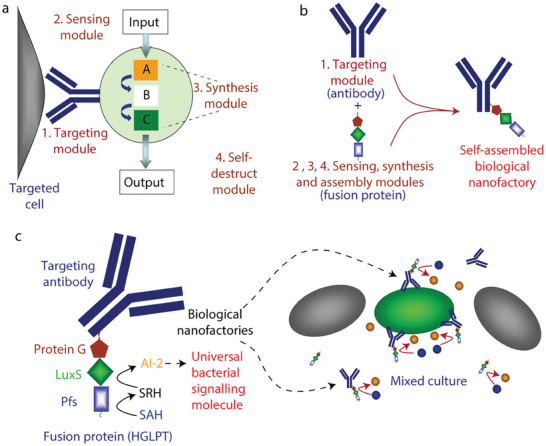
Engineered biological nanofactories trigger a quorum sensing response in targeted bacteria. a) Components of a conceptual biological nanofactory comprising four functional modules. b) Components of the practical demonstration of a biological nanofactory: targeting module (cell targeting antibody), and sensing, synthesis, and assembly modules (fusion protein). c) The nanofactories self‐assemble when protein G of fusion protein HGLPT binds to the Fc region of the targeting antibody. Following their addition to bacterial cultures, nanofactories bind specifically to the targeted bacteria (green circle), synthesize and deliver AI‐2 (yellow circles) at their cell surfaces and trigger the quorum sensing response. Reproduced with permission.^[^ ^169^ ^]^ Copyright 2010, Springer.

**Figure 6:**
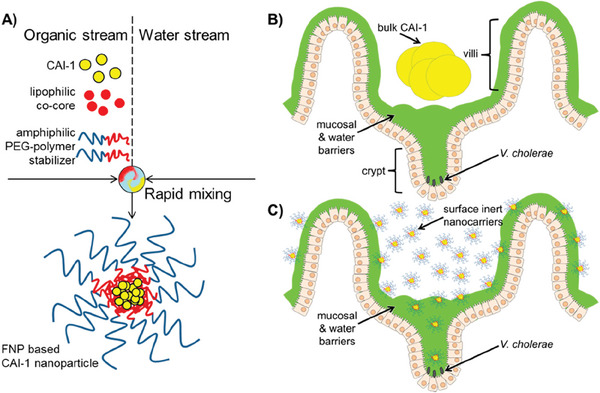
Flash NanoPrecipitation and drug delivery of CAI‐1. A) CAI‐1 nanoparticles decorated with a polyethylene glycol surface (PEG) can be formed by rapidly precipitating CAI‐1 in the presence of amphiphilic PEG diblock copolymers and lipophilic co‐core materials. B) Hydrophobic bulk CAI‐1 cannot penetrate the mucus that covers crypts in the small intestine where V. cholerae reside during infection. C) Delivery of CAI‐1 in small PEG covered nanoparticles can allow CAI‐1 to directly penetrate mucus layers or become rapidly solubilized into bile micelle carriers that can also penetrate intestinal barriers. Reproduced with permission.^[^ ^24^ ^]^ Copyright 2015, American Chemical Society.

Second, nanomaterials can positively regulate bacterial QS by delivering key QS components such as QS signal synthase and QS signal molecules. The nanofactory consists of a bacteria capture module and a QS signal synthesis module; of these, the capture module is responsible for combining with the bacterial surface and the synthesis module is responsible for providing bacteria with QS signal synthase. Consequently, the nanofactory enables local synthesizes of high concentrations of QS signal molecules to regulate bacterial group behavior.^[^ ^168^, ^169^, ^170^ ^]^ Studies have showed that by attaching AI‐2 synthetases (including S‐adenosylhomocysteine nucleosidase [Pfs] and LuxS) to magnetic chitosan‐mag NPs, nanofactory with the ability to synthesize AI‐2 can be successfully prepared. This nanofactory can not only activate the specific transcriptional response of AI‐2, but also be directed and recovered via an external magnetic field.^[^ ^168^ ^]^ Further studies have found that the signal synthesis module is more efficient at assembling Pfs and LuxS with fusion proteins than at using them separately, probably due to the shorter distance between the two enzymes in the fusion protein.^[^ ^170^ ^]^ It is notable that capturing bacteria based on the charge characteristics of chitosan is non‐specific, which can chemically graft appropriate antibodies to give nanofactories the ability to specifically bind to target bacteria (**Figure** **5**).^[^ ^169^ ^]^ Aside from delivering QS signal synthase, nanomaterials can regulate bacterial QS by directly delivering QS signal molecules, which is mainly applied to bacteria that inhibit virulence by QS system. Studies have shown that by using polystyrene‐block‐PEG (PS‐b‐PEG) as the stabilizer and vitamin E as a co‐core component, CAI‐1‐loaded NPs (CAI‐1 NPs) can be synthesized to deliver the QS signal molecule CAI‐1. CAI‐1 NPs can effectively inhibit toxin secretion and biofilm formation by providing exogenous CAI‐1 to activate the QS system of *V. cholerae* (**Figure** **6**).^[^ ^24^ ^]^

Lastly, nanomaterials (such as graphene oxide) can use their surface topologies to directly activate bacterial QS signal receptors, thereby manipulating bacterial group behaviors.^[^ ^171^ ^]^ The key of this strategy is to improve the recognition efficiency of nanomaterials to bacterial surface QS receptors, which can be achieved by producing highly ordered surface topologies using nanolithographic techniques and promoting the strong multivalent interaction between nanosurface topologies and QS signal receptors in a biomimetic and molecularly selective manner.^[^ ^172^ ^]^

## 4 Optimization Strategies of the Quorum Sensing Regulation Activity of Nanomaterials

**Figure 7:**
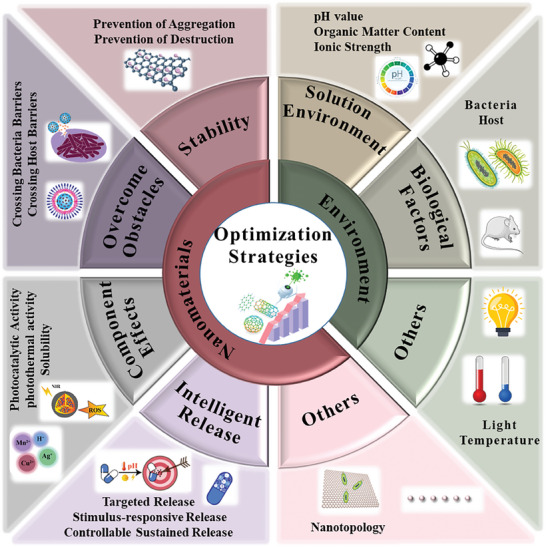
Optimization strategies of the quorum sensing regulation activity of nanomaterials.

Enhancement of the QS regulatory activity of nanomaterials to increase their practical availability is one of the key exploration areas for improving the performance of such nanomaterials at this stage. This section combines the two key factors of “subject‐environment” (i.e., nanomaterials and environmental factors) that affect the QS regulatory activity to propose specific optimization strategies for enhancing the QS regulatory activity of nanomaterials, in order to provide reference for the rational modification and design of nanomaterials (**Figure** **7**) (**Table** **2**).

**Table 2:** Factors affecting QS regulatory activity of nanomaterials

Influencing factors	Nanomaterials	Bacteria	Experimental grouping	Dose	Index	Result	Ref.
Type	ZnO NPs、TiO_2_ NPs and Ag NPs	*C. violaceum*	ZnO NPs、TiO_2_ NPs and Ag NPs	0, 10, 100, 250, 500 ppm	violacein production	Ag NPs have the strongest anti‐QS ability, followed by ZnO NPs, and TiO_2_ NPs are the weakest. ZnO NPs disturb signal perception and response, whereas TiO_2_ NPs and Ag NPs affect signal synthesis.	[14]
Type	Se NPs and Te NPs	*C. violaceum* and *P. aeruginosa*	Se NPs and Te NPs	0, 10, 50, 100, 250 ppm	violacein production and colony‐forming unit	Te NPs have the stronger anti‐QS ability than Se NPs. Se NPs interrupt signal synthesis whereas Te NPs affect signal perception and response.	[10]
Type	nine NPs (Ag, Fe, ZnO, TiO_2_, SiO_2_, Fe_2_O_3_, SWCNTs, GO, and C_60_	*P. Aeruginosa*	, nine NPs, Ag^+^, Fe^2+^ and Fe^3+^	100 µg L^−1^	signal release, receptor activity, protease production,biofilm biomass and biofilm morphology	Ag and GO increased the N‐3‐oxo‐dodecanoyl homoserine lactone (3OC_12_‐HSL) concentration, protease production and biofilm formation, but didn‘t influent N‐butanoyl homoserine lactone. Fe ENPs increased the 3OC_12_‐HSL concentration,but didn‘t increase protease production or biofilm formation.	[173]
Type	PEIMnF and PEINF NPs	*S. aureus, E. coli, C. albicans* and *C. violaceum*	polyethyleneimine‐coated magnetite (PEIMnF) and nickel ferrite (PEINF NPs)	10, 5, 2.5, 1.25, 0.625 mg mL^−1^; 1, 1/2, 1/4, 1/8, 1/16,1/32 minimum inhibitory concentration (MIC)	antibiofilm activity, violacein formation, swimming, and swarming motility	Only PEINF showed a violacein inhibition. PEINF showed better inhibition in swimming and swarming inhibition.	[77]
Shape	Ag NPs and Ag NRs	*P. aeruginosa*	Ag NPs and Ag nanorods (NRs)	1, 20,25 mg L^−1^; 1, 30, 300 mg L^−1^	optical density 600, live/dead ratio, ROS and nitrate levels, transmission electron microscopy images of membrane damage, transcript profiles of related genes	Ag NPs showed a stronger inhibitory effect and induced greater transcriptional responses.	[56]
Shape	Ag NPs	*C. violaceum*	40 and 60 nm	4 mgAg L^−1^	violacein production	Smaller Ag NPs produce a more significant alteration of QS system.	[14]
Shape	ZN1 and ZN2	*C. violaceum* and *P. aeruginosa*	ZnO nanospikes with 4 weeks incubation periods (ZN1) and ZnO nanospikes with 6 weeks incubation periods (ZN2)	25, 50, 100, 200 µg mL^−1^	violacein production, virulence production, transcriptional activity, and biofilm formation	ZN2 had a higher inhibitory action on the virulence productivity and biofilm formation than ZN1.	[174]
Dose	aTiO_2_‐NPs	freshwater biofilms	0.1 mg L^−1^ and 10 mg L^−1^	0.1 mg L^−1^ and 10 mg L^−1^	biofilm amount and activity, microstructure of biofilm, the contents of AHL and AI‐2	Low levels of TiO_2_‐NPs advanced planktonic cellcol onization and biofilm formation and maturation. High concentration inhibited and delayed biofilm development.	[175]
Dose	ZnO NPs、TiO_2_ NPs and Ag NPs	*C. violaceum*	0, 10, 100, 250, and 500 ppm	0, 10, 100, 250, and 500 ppm	violacein production and cellular viability	The QS regulation ability was concentration‐dependent. ZnO NPs interrupt QS between 10 mgZn L^−1^ and 100 mgZn L^−1^. Up to 500 mgTi L^−1^ of TiO_2_ NPs was required to regulate QS. As low as 4 mgAg L^−1^ of Ag NPs was required to regulate QS.	[14]
Dose	ZnO NPs	*P. putida*	0, 100, 200, 300, 400, 500 mg L^−1^; 0, 0.5, 250 mg L^−1^; 0.5, 250 mg L^−1^	0, 100, 200, 300, 400, 500 mg L^−1^; 0, 0.5, 250 mg L^−1^; 0.5, 250 mg L^−1^	bacterial growth, biofilm development, ROS and malondialdehyde levels, and expression of biofilm‐related genes	Low concentrations of NPs promoted bacterial growth and biofilm formation, while high concentrations inhibited biofilm formation.	[103]
Dose	Ag NPs‐MK	*C. violaceum, S. marcescens* and *P. aeruginosa*	0, 0.5, 1, 2, 4 µg mL^−1^; 0, 1, 2, 4, 8 µg mL^−1^; 1/16, 1/8, 1/4, 1/2MIC;	0, 0.5, 1, 2, 4 µg mL^−1^; 0, 1, 2, 4, 8 µg mL^−1^; 1/16, 1/8, 1/4, 1/2MIC;	MIC, violacein production, virulence production, biofilm inhibition	Ag NPs‐MK inhibited the virulence factors and bioflms in a dose‐dependent manner at sub‐MICs.	[176]
Dose	CuO NPs	seeded sludge	1, 5, 10, 20, 50 mg L^−1^	1, 5, 10,20, 50 mg L^−1^	microbial community detection, ammonia removal efficiency, nitrite accumulation rate, ammonia removal rate, nitrogen removal rate, and total nitrogen removal efficiency	5 mg L^−1^ CuO NPs contributed to the survival of Anammox bacteria. 50 mg L^−1^ NPs suppressed the Anammox bacteria.	[80]
Structure	Ag NPs	*C. violaceum*	polyvinylpyrrolidone capping and citrate capping	4 mgAg L^−1^	violacein production	Ag NPs with citrate capping have stronger QS regulatory activity.	[14]
Structure	pSWCNTs‐Ag	*E. coli, S. aureus, S. pyogenes* and *S. typhimurium*	silver coated single walled carbon nanotubes (SWCNTs‐Ag) and pegylated SWCNTs (pSWCNTs‐Ag)	62.5, 31.25, 15.12 or 7.8 µg mL^−1^; 62.5, 31.25 µg mL^−1^; 31.25, 15.6 µg mL^−1^; 50, 25, 12.5, 6.25, 0 µg mL^−1^; 12.5 µg mL^−1^	cytotoxicity and antibacterial activity	pSWCNTs‐Ag are more efficient than plain SWCNTs‐Ag. pSWCNTs‐Ag are non‐toxic to human cells at bactericidal concentration.	[177]
Solution environment	QSINPs	*P. aeruginosa* and *E. coli*	acidic pH (4.0) and neutral pH (7.0)	600 mg kg^−1^; sub‐MIC; MIC; 2MIC;	release profile of QSI	At pH 7.0, burst release of QSI was observed, indicating 61.6% release within 3 h and 100% release in 12 h. At pH 4.0, sustained release of QSI was observed indicating 16.9% release in 3 h and 100% release in 16 h.	[39]
Illumination	TiO_2_ NPs	*C. violaceum*	ultraviolet (UV) exposure time of 0, 1, 2 h	500 mgTi L^−1^ and 100 mg Ti L^−1^	violacein production	The QS regulation ability of TiO_2_ NPs was not affected by UV irradiation.	[14]
Biological environment	AgNPs‐MK	*C. violaceum, S. marcescens, P. aeruginosa*	*C. violaceum, S. marcescens, P. aeruginosa*	4 µg mL^−1^, 8 µg mL^−1^ and 8 µg mL^−1^	microscopic analysis of bioflms	The biofilms of *C. violaceum*, *S. marcescens*, and *P. aeruginosa* were reduced by 87.39, 81.54, and 71.34%, respectively.	[176]
Biological environment	bio‐AgNPs	10 clinical isolates, 5 environmental isolates and 2 reference strains	10 clinical isolates, 5 environmental isolates and 2 reference strains	½ MIC (7.81–31.25 µM)	motility, biofilm formation	sub‐MICs of bio‐AgNPs increased biofilm formation and motility of some *P. aeruginosa*.	[178]
Biological environment	bio‐Ag NPs	*P. Aeruginosa*	*P. Aeruginosa PAO1, PA14*	0, 7.81, 15.62, 31.25 µM	MIC, growth curve, production of virulence, the expression of QS‐related genes	Sub‐MICs of bio‐Ag NPs reduced the motility and virulence in PAO1, but stimulated motilities and virulence in PA14. Bio‐Ag NP increased the expression of QS genes in PAO1 and PA14.	[179]
Biological environment	ZnO NPs	two laboratory strains, six clinical strains, a furanone C‐30 resistant strain, two PA14 gallium resistant mutants, a PA14 C‐30 resistant mutant and four environmental isolates.	PAO1, PA14, INP‐37, INP‐42, INP‐58 M, INP‐57 M, INP‐58R, 5U, INP‐42R, TC5, E12, MexR, M10, 148, ID4365, IGB83	1 mmol L^−1^	production of virulence, biofilm formation and hydrophobicity	ZnO NPs decreased virulence production and biofilm formation for most of the strains. ZnO NPs have a broad spectrum for the QQ activity.	[180]
Others	aTiO_2_‐NPs	freshwater biofilms	Control, anatase‐TiO_2_ NPs (TiO_2_‐A), aged TiO_2_‐A, rutile‐TiO_2_ NPs (TiO_2_‐R), aged TiO_2_‐R	0.1 mg L^−1^ and 10 mg L^−1^	biofilm amount and activity, microstructure of biofilm, contents of AHL and AI‐2	Aging didn't change the crystal structure, but deactivate the photo‐activities and increase stability.	[175]

### 4.1 Optimization of Nanomaterials

The physicochemical properties of nanomaterials are closely related to their application performance. At present, the nanomaterial‐related factors that affect the application performance of nanomaterials with QS regulatory activity mainly include the following: first, they lack sufficient stability and are prone to aggregation or destruction during storage and delivery, leading to loss of efficacy.^[^ ^181^ ^]^ Second, their ability to cross delivery barriers is limited; hence, nanomaterial dosage increase is needed to meet the drug concentration requirements of the target site. However, this may cause biosafety issues.^[^ ^182^ ^]^ Third, the preparation cost of nanomaterials is relatively high, and enhancing the QQ activity of the effector component can effectively reduce nanomaterial dose and application costs while ensuring efficacy.^[^ ^183^ ^]^ Lastly, the biological environment in vivo is much more complicated compared with that under laboratory conditions; however, the functional design of nanomaterials is relatively simple at the current stage, and hence the ideal results of the experimental stage cannot be achieved in the clinical setting.^[^ ^184^ ^]^ Therefore, this section combines factors that affect the physicochemical properties of nanomaterials to elaborate the existing strategies for optimizing the QS regulatory activity of nanomaterials from the following four aspects.

#### 4.1.1 Improvement of the Stability of Nanomaterials

The stability of nanomaterials is essential for their QS regulatory activity.^[^ ^185^ ^]^ During the storage process, due to the characteristics of large specific surface area and high surface energy, nanomaterials are often in an energetically unstable state, and thus tend to aggregate. The increased particle size deteriorates aggregated nanomaterial properties and sharply decreases their QS regulatory activity.^[^ ^38^ ^]^ During the delivery process, nanomaterials with QS regulatory activity are vulnerable to environmental damage (such as fluid percussion and enzymatic degradation), resulting in drug efficacy loss.^[^ ^29^ ^]^ Therefore, improving nanomaterial stability to prevent aggregation or destruction during storage and delivery is an important strategy to optimize the QS regulatory activity of nanomaterials; among them, the prevention of aggregation is more widely studied.

**Figure 8:**
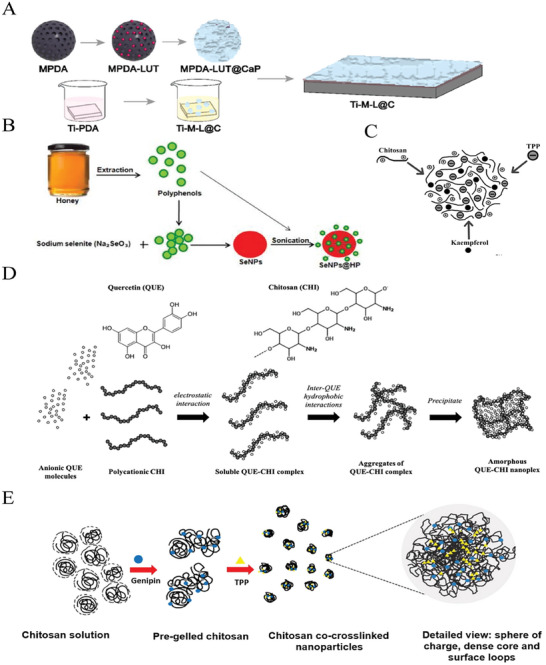
Synthesis methods of nanomaterials with QS regulatory activity A) Schematic diagram the preparation process of Ti‐M‐L@C substrate. Reproduced under the terms of the Creative Commons Non‐Commercial, No Derivatives (CC BY‐NC‐ND) license.^[^ ^25^ ^]^ Copyright 2022, The Authors. B) Pictorial representation for the biofabrication of scaffold of SeNPs and HP (SeNPs@HP). Reproduced under the terms of the CC BY license.^[^ ^27^ ^]^ Copyright 2017, The Authors. C) Schematic illustration of kaempferol encapsulated chitosan nanoparticles. Reproduced with permission.^[^ ^92^ ^]^ Copyright 2016, Elsevier. D) Schematics of the quercetin (QUE)‐chitosan (CHI) nanoplex formation. Reproduced under the terms of the CC‐BY license.^[^ ^50^ ^]^ Copyright 2021, The Authors. E) Model of formation of PC‐NPs showing the two steps of crosslinking with GNP and TPP and details of the structure and surface topology of the furnished NPs. Reproduced with permission.^[^ ^192^ ^]^ Copyright 2020, Elsevier.

To solve the problem of easy aggregation of nanomaterials with QS regulatory activity during storage, existing studies mainly improved the stability of nanomaterials by surface functional modification, doping modification, and synthesis method improvement to prevent nanomaterial aggregation. Surface functional modification is the most commonly used strategy.^[^ ^64^ ^]^ Various organic and inorganic molecules (such as polymers or silica) are used to functionalize the surface of nanomaterials by physical adsorption or chemical attachment, and due to the steric hindrance effect, the dispersion stability of nanomaterials is improved.^[^ ^186^ ^]^ For example, when the silica layer is grafted on the surface of yttrium oxide NPs, silanol (Si‐OH) groups covering its surface can easily form a colloidal solution in aqueous media by hydrogen bonding.^[^ ^66^ ^]^ Chemical attachment produces better stability than physical adsorption, and monolayer surface modification can often be obtained. When achieving surface functionalization of nanomaterials to improve their dispersion stability, the structural characteristics of the original nanomaterials are retained to the greatest extent.^[^ ^187^ ^]^ Second, doping modification is an important strategy to improve nanomaterial dispersion stability.^[^ ^33^ ^]^ Different from the steric hindrance effect of surface functional modification, doping modification mainly improves the energy barrier of the reaction through covalent bonding between doping components and nanomaterial matrix, so as to reduce the chemical activity of nanomaterials and enhance their stability as well as other properties.^[^ ^188^ ^]^ Therefore, the doping composition is the key to the optimization strategy; the types of doping composition, their distribution in the matrix, and the interaction mechanism with the matrix are the main factors involved in this strategy.^[^ ^189^ ^]^ Interestingly, surface functional modification and doping modification not only optimize the QS regulatory activity of nanomaterials by improving the dispersion stability, but also directly enhance the QS regulatory ability of nanomaterials. However, their nanomaterial activity optimization mechanisms are different; the former may be related to the formation of the core/shell structure, whereas the latter may be related to lattice defect sites.^[^ ^66^, ^190^ ^]^ Finally, different synthesis methods can lead to significant differences in the ultrastructure of materials, resulting in a wide range of different physicochemical and biological properties of the materials (**Figure** **8**).^[^ ^174^ ^]^ Therefore, the more stable nanomaterials can be obtained by constantly optimizing and improving the synthesis method. Ionically crosslinked chitosan‐based NPs synthesized by physical crosslinking methods have a strong and stable QQ activity; nevertheless, these NPs have a poor colloidal stability, and they are prone to aggregation, which limits their wide application as QSIs.^[^ ^191^ ^]^ Further studies have found that the covalent co‐crosslinking chitosan NPs synthesized by chemical crosslinking methods can improve nanomaterial stability to tolerate pH, temperature change, as well as biological and mechanical degradation, although their QQ ability is weakened. To further improve the synthesis process, chitosan and chemical crosslinking agents were crosslinked under the condition close to the critical gelling, followed by ionotropic formation of NPs in the presence of the ionic crosslinking agent. This resulted in NPs displaying more stable physicochemical properties while maintaining strong QQ and antibacterial activities.^[^ ^192^ ^]^ Some studies have also demonstrated that biosynthetic nanomaterials have higher stability, mainly because the biological capping agent on their surface provides a protective layer to prevent oxidation and aggregation.^[^ ^193^ ^]^ Moreover, the ionic strength of the solvent affects nanomaterial stability during the synthesis process.^[^ ^194^ ^]^ Chitosan NPs synthesized in salt solution are smaller, denser, more stable, and have a narrower polydispersity compared with those synthesized in pure water.^[^ ^191^ ^]^

Nanomaterials with QS regulatory activity are vulnerable to environmental damage during application. Physical isolation and bonding fixation can be used to protect nanomaterials and maintain their potency.^[^ ^30^, ^194^ ^]^ Different protection strategies are suitable for different application environments, and the appropriate design strategy can be chosen based on the needs. The shell structure with physical isolation function can be designed to protect the nanomaterials from biological reactions in the host (such as proteolytic processes catalyzed by proteolytic enzymes) to maintain the potency.^[^ ^39^ ^]^ For example, the lipid bilayer structure can be used as a physical protective barrier to limit the entry of proteases, such that nanomaterials can be used in proteolytic enzyme‐rich infectious environments (such as open trauma).^[^ ^195^ ^]^ Under shaking conditions, the QQ enzyme simply adsorbed on the nanomaterial surface is easily detachable, which causes structural disruption and inactivation.^[^ ^31^ ^]^ QQ enzymes that are chemically bonded with nanomaterials do not separate and denature even when subjected to vigorous mechanical shaking.^[^ ^196^ ^]^ In previous works, nano‐filtration membranes with QQ function have been prepared by applying the enzyme adsorption, precipitation, and crosslinking (EAPC) protocol. The nano‐filtration membrane has good stability due to the formation of many chemical crosslinking bonds between QQ enzymes and carbon nanotubes. Under water flow conditions, the enzyme‐loading ability of EAPC membranes is 5.6 times higher than that of adsorption membranes (enzyme is simply adsorbed on the nanomaterial surface).^[^ ^29^ ^]^ Some materials (such as gold [Au]) are more conducive to the stabilization of biomolecules (such as QQ enzymes and natural QSIs). In the future, coating Au on nanomaterial surfaces to obtain biocomposite surfaces, and the application of these surfaces in bacterial QS regulation will be a promising strategy.^[^ ^138^ ^]^

#### 4.1.2 Improvement of the Transport of Nanomaterials across Barriers

The defensive barrier of bacteria and the complex in‐vivo environment of the host are important factors that reduce nanomaterial drug delivery efficiency, thereby affecting nanomaterial QS regulatory activity.^[^ ^24^ ^]^ Enhancing the ability of nanomaterials to cross the abovementioned drug delivery barriers will greatly improve their QS regulatory activity.

**Figure 9:**
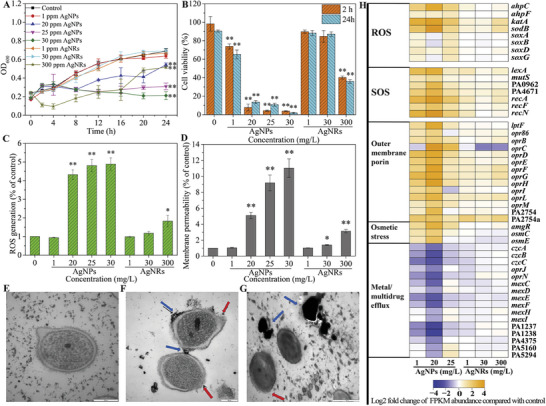
Inhibitory effects of AgNPs and AgNRs on P. aeruginosa PAO1. A) Growth profiles in terms of OD600, B) live/dead ratio, C) the intracellular ROS level, and D) the cell membrane permeability of PAO1 under the exposure to AgNPs/AgNRs. Images of the (E) non NPs‐treated control cells and of cells in the presence of F) 20 mg L^−1^ AgNPs and G) 300 mg L^−1^ AgNRs by transmission electron microscopy (TEM). For E and F: bar = 200 nm, and for G: bar = 500 nm. Blue arrows indicate AgNPs/AgNRs, red arrows indicate membrane damage. H). The transcript profilesof PAO1 responses related to oxidative stress, SOS response, outer membrane porin, osmotic stress, and efflux systems under the exposure of AgNPs/AgNRs. Reproduced with permission.^[^ ^56^ ^]^ Copyright 2019, Elsevier.

The bacterial defensive barrier mainly includes biofilm, cell membrane, and cell wall. Currently, the ability of nanomaterials to cross the bacterial defensive barriers is enhanced by improving the nanomaterial barrier penetration ability and weakening the bacterial barrier defense ability; the former is more common.^[^ ^33^ ^]^ The penetration ability of nanomaterials can be enhanced by adjusting their basic physical and chemical parameters (such as surface charge, roughness, size, and shape), and thereby optimizing their QS regulatory activity.^[^ ^197^ ^]^ First, the surface charge significantly affects the adsorption of nanomaterials to biofilms and bacterial membranes, and nanomaterials with positively‐charged surfaces are more likely to be adsorbed by negatively charged bacterial barriers.^[^ ^51^, ^129^ ^]^ Surface modification of nanomaterials to carry a positive charge may promote their adhesion to bacterial and biofilm surfaces, thereby enhancing their permeability potential.^[^ ^73^ ^]^ Chitosan with positively‐charged amino groups is the most popular surface modification component. It has the characteristics of biofilm adhesion and hydrophilicity, which enables various poorly absorbable drugs to cross defensive barriers.^[^ ^64^ ^]^ Moreover, chitosan can destabilize the bacterial cell membrane, thereby enhancing the drug efficacy.^[^ ^198^ ^]^ However, a previous study has shown that the net negative charge of NPs can prevent them from accumulating on biofilm surfaces, and when the NPs are weakly alkaline and positively charged, they cannot penetrate the biofilm to reach its core where resistant bacterial subpopulations exist.^[^ ^199^ ^]^ Therefore, it is still a challenge to reasonably design the surface charge of nanomaterials to achieve the best balance between adsorption and penetration of bacterial defensive barriers. Second, smaller sizes are more conducive for bacterial defense barrier penetration by nanomaterials, thereby better exerting QS regulatory functions.^[^ ^56^, ^193^ ^]^ Polyacrylic acid‐coated iron oxide NPs are reportedly smaller than uncoated NPs, which is more convenient for internalization into bacteria to enhance QS regulation.^[^ ^200^ ^]^ Besides, nanomaterial shape is an important optimization parameter that can be used to promote penetration across bacterial defense barrier (**Figure** **9**). At present, the nanomaterials used for directly regulating bacterial QS are mostly spherical and rod‐shaped, as they may be more conducive for cellular uptake and internalization.^[^ ^201^ ^]^ Further studies have found that particles with an aspect ratio of 2.1–2.5 are taken up faster and in larger quantities, compared with spheres as well as shorter and longer rods.^[^ ^202^ ^]^ Although spherical or rod‐shaped nanomaterials have good QS regulatory activity, the influence of shape‐related factors on biosafety, pharmacokinetics, and other parameters should be considered in the context of in‐vivo applications of such nanomaterials. For example, compared with spherical or rod‐shaped Au NPs, Au nanotubes, nanostars, and nanoflowers have longer in‐vivo circulation time and lower biotoxicity.^[^ ^203^ ^]^ When developing novel nanomaterials with QS regulatory activity, future studies should not be limited to spherical or rod‐shaped nanomaterials. Practically, different nanomaterial shapes can be designed to enhance their QS regulatory role in vivo. Few studies have evaluated the effect of surface roughness (compared with the abovementioned related parameters) on nanomaterial penetration across bacterial defensive barriers. Although a rough nanomaterial surface is conducive for bacterial adsorption, an increase in surface roughness may cause the adsorption of more proteins in the medium on the nanomaterial surface, thereby weakening their adhesion to bacteria.^[^ ^69^ ^]^ It should be noted that all parameters are interrelated, and the QS regulatory activity is the result of a delicate balance between the parameters; considering only a single parameter may produce results that are contrary to the design concept. Apart from improving the barrier penetration ability of nanomaterials, other drugs can be used in concert with nanomaterials to destroy the biofilm and bacterial cell wall/membrane structures, thereby weakening the barrier defense of these structures and promoting the transport of nanomaterials across barriers. For example, terpenes and terpenoids can damage the bacterial cell wall by interacting with phenyl groups of critical proteins in the cell wall.^[^ ^204^ ^]^ Berberine can weaken the cell wall/membrane resistance of methicillin‐resistant *Staphylococcus aureus* to drugs by reducing the lipid content of its cell membrane and up‐regulating the genes related to cell wall hydrolysis.^[^ ^205^ ^]^

Along with the defensive barrier of bacteria, the complex in‐vivo environment of the host (such as gastric acidic environment, intestinal and pulmonary mucous layers, and hepatic first‐pass effect) acts as another drug delivery barrier of nanomaterials.^[^ ^24^ ^]^ Enhancing the ability of nanomaterials to overcome the complex host environment for drug delivery is another important direction to optimize the QS regulatory activity of nanomaterials.^[^ ^40^ ^]^ However, due to the complexity of influencing factors in vivo, the difficulty of continuous visual observation using in‐vivo models, and the difficulty of constructing in vitro models simulating real in‐vivo environments, the development of this kind of nanomaterials is still at an early stage. Among the various strategies being pursued to this end, the most common optimization method involves coating the surface with hydrophilic polymer to improve nanomaterial hydrophilicity and mucous permeability.^[^ ^206^ ^]^ Using hydrophilic PS‐b‐PEG as a stabilizer and vitamin E as a co‐core component, a kind of nanomaterial that encapsulates various QS regulators has been synthesized. This nanomaterial can use bile in the small intestine as a trigger for a rapid drug dissolution and release, thereby causing lipophilic drug release through the mucus barrier.^[^ ^24^ ^]^

#### 4.1.3 Enhancement of the Component Effects of Nanomaterials

The QQ ability of nanomaterials is closely related to their types.^[^ ^77^ ^]^ Different kinds of nanomaterials have different modes of action, and thus their mechanism and ability to quench bacterial QS are also different.^[^ ^14^ ^]^ Targeted optimization methods should be selected for specific types of nanomaterials.

Some metal‐based nanomaterials perform QQ functions mainly by dissolving metal ions.^[^ ^193^ ^]^ The solubility of this kind of nanomaterials is mainly enhanced in three ways (size, shape, and surface modification), which optimizes their QQ activity. First, the QQ ability of these nanomaterials can be enhanced by reducing the size. The decrease in size can increase the specific surface area and formation enthalpy of nanomaterials, thereby significantly increasing their dissolution rate and equilibrium concentration.^[^ ^207^ ^]^ Second, the QQ activity of the nanomaterials can be effectively enhanced by designing them with shapes that are more conducive for dissolution. For example, the dissolution rate of spherical NPs is much faster than that of rod‐shaped and spindle‐shaped NPs.^[^ ^208^ ^]^ Finally, surface modification using materials with little effect on solubility can be applied to enhance the QQ activity of the nanomaterials. Surface modification is applied for most existing nanomaterials to obtain better targeting and stability; however, due to the physical barrier, surface modification significantly affects nanomaterial solubility.^[^ ^209^ ^]^ Preferentially selecting materials with simpler structures for surface modification minimizes their adverse effects on nanomaterial solubility. For example, because the chain length and structural complexity of PEG are lower than that of polydiallydimethylammounium chloride (PDDA), the dissolution rate of PDDA‐coated CdSe/ZnS QDs is much higher than that of PEG‐coated CdSe/ZnS QDs.^[^ ^210^ ^]^

Photocatalytic nanomaterials perform QQ function mainly through the generation of ROS. The QQ activity is closely related to the photocatalytic performance, which is mainly affected by the energy band structure, morphology, defects, and size.^[^ ^211^ ^]^ Nanomaterials with stronger photocatalytic activity can be directly selected to obtain stronger QQ activity. For example, compared with other crystalline, anatase‐phase TiO_2_ NPs have the strongest photocatalytic activity, probably due to their small grain size. This reduces the time for photogenerated electrons and holes to diffuse from the inside to the surface of TiO_2_, thereby reducing their recombination probability in TiO_2_ and enhancing the photocatalytic activity.^[^ ^28^ ^]^ Besides selecting types of nanomaterials with stronger photocatalytic activity, the photocatalytic activity of nanomaterials can be enhanced by material modification, resulting in QQ activity optimization. The specific modification methods are as follows: (1) improving the separation efficiency of photogenerated carriers on the surface of nanomaterials by precious metal deposition;^[^ ^212^ ^]^ (2) enhancing the charge separation efficacy of nanosystems by coupling with semiconductors and expanding the spectral absorption range;^[^ ^213^ ^]^ (3) suppressing the recombination of electrons and holes and extending the carrier lifetime by doping metal ions to cause lattice defects or alter crystallinity;^[^ ^214^ ^]^ and (4) directly enhancing the photocatalytic activity of nanomaterials by increasing the number of surface hydroxyl groups.^[^ ^215^ ^]^

**Figure 10:**
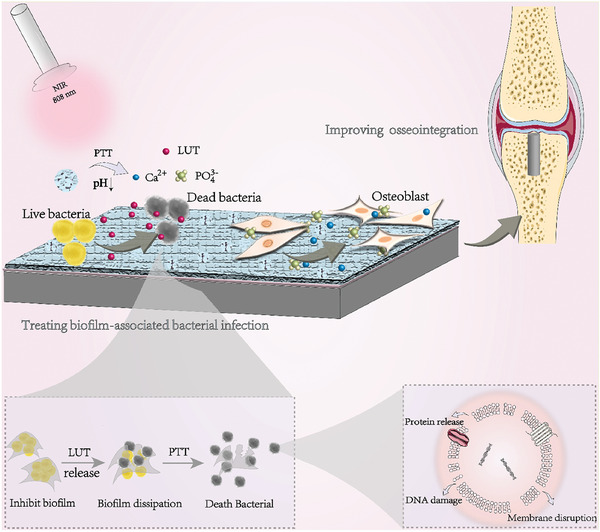
Schematic diagram of combining PTT and quorum‐sensing‐inhibition strategy for improving osseointegration and treating biofilm‐associated bacterial infection of Ti‐based implant. Reproduced under the terms of the CC BY‐NC‐ND license.^[^ ^25^ ^]^ Copyright 2022, The Authors.

Although studies have reported bacterial QS inhibition by photothermal nanomaterials, this field is still in an early stage; more comprehensive and in‐depth studies are needed to develop photothermal nanomaterials with high photothermal conversion efficiency, broad‐spectrum irradiation, and good biosafety (**Figure** **10**).^[^ ^25^, ^78^ ^]^ Among them, improving photothermal conversion efficiency is the key to ameliorate the therapeutic efficacy of photothermal therapy (PTT). A simple strategy has been designed to improve the efficiency of photothermal conversion by changing NPs from aggregated state to dispersed state. Compared with NPs in the aggregated state, those in the dispersed state can rotate freely, thereby achieving nonradiative dissipation through twisted intramolecular charge transfer effect and producing an excellent photothermal conversion efficiency.^[^ ^216^ ^]^ In addition, the development of photothermal nanomaterials with broad‐spectrum irradiance can simplify the usage conditions and broaden the application range. For example, Au substrate coupled with Ag nanocubes can produce large concentrations of hot electrons in a broad spectral range from the ultraviolet to NIR regions of the spectrum.^[^ ^217^ ^]^ It should be noted that PTT cannot blindly pursue high heat generation, because excessively high temperatures can damage surrounding normal tissues. However, the relatively low temperature of mild PTT has limited QQ function; hence, combination with other therapies is required to construct a synergistic treatment system for antibacterial therapy.^[^ ^218^ ^]^ Increasing the temperature can augment the permeability of bacterial cell membranes and promote bacterial absorption of other drugs. Meanwhile, the use of other drugs can reduce bacterial heat tolerance, thereby enhancing PTT efficacy.^[^ ^219^ ^]^ In addition to combination treatment, targeted selectivity, remote controllability, and minimal invasiveness may be feasible directions for the future development of photothermal nanomaterials with good biological safety.

Current research on the QS regulatory activity of nanomaterials focuses on metal‐based nanomaterials and organic nanomaterials; less attention is paid to inorganic and non‐metallic nanomaterials with higher biological safety. However, it has been shown that SeNPs and TeNPs with lower biotoxicity also have QQ functions, which can significantly inhibit the production of violet pigment by *Chromobacterium violaceum* and reduce the biomass of *P. aeruginosa* biofilm by 80%.^[^ ^10^ ^]^ In the future, more attention should be paid to the QQ activity and optimization of inorganic non‐metallic nanomaterials.

#### 4.1.4 Enhancement of the Intelligent Release Ability of Nanomaterials

Nanomaterials with QS regulatory activity exhibit off‐target effects and burst release phenomena in complex practical application environments, leading to low bioavailability of drugs and serious toxic side effects.^[^ ^220^ ^]^ Enhancing the intelligent release ability of nanomaterials (including targeted, stimulus‐responsive, and controllable sustained release) will effectively solve the abovementioned problems, thereby greatly optimizing the QQ activity of nanomaterials in practical applications. Among them, targeted release solves the problem of where the nanomaterials are released; stimulus‐responsive release solves the problem of when they are released; while controllable sustained release solves the problem of release dosage and time. With ongoing advancements in research, the intelligent release ability of nanomaterials is no longer limited to a single function. Intelligent drug delivery nanosystems with more complex and diverse functions are being developed; this area is expected to become a research hotspot in the future.

Designing targeted nanomaterials that directly target bacterial biofilms at the infected site can promote high‐dose absorption of drugs at the target site.^[^ ^39^ ^]^ Targeting designs include passive targeting and active targeting designs. Passive targeting designs are mainly achieved by enhancing the penetration and retention of nanomaterials at the infected site, which is related to the physicochemical properties of the nanomaterials (such as magnetic property and sharp edge).^[^ ^221^ ^]^ Active targeting designs are based on the attraction of bacteria to nanomaterials, such as electrostatic interactions between cells and nanomaterials and ligand‐receptor or antibody‐antigen recognition.^[^ ^222^ ^]^ Active targeting designs are mainly achieved through nanomaterial surface modification. Compared with targeting designs based on non‐specific electrostatic interactions, specific interactions based on complex ligand‐receptor or antibody‐antigen recognition have the smallest off‐target effects on host cells. However, it should be noted that modifying the targets by drug‐resistant bacteria may reduce these specific recognitions.^[^ ^223^ ^]^ Prioritization of the design and development of intelligent nanomaterials targeting high expression levels of bacterial surface receptors or pathogen‐specific antigens is recommended. Interestingly, cell‐derived membranes have become a promising coating material for targeted delivery due to their unique advantages such as targeting ability, bacterial toxin neutralization, immune escape, and blood circulation time prolongation.^[^ ^2^ ^]^ When incubated with bacteria, the specific bacterial related receptors of these cells are correspondingly upregulated, which facilitates precise and rapid bacterial recognition.^[^ ^224^ ^]^

Designing stimuli‐responsive nanomaterials can achieve on‐demand drug release at the site of infection. The low pH, bacterial toxins (such as perforin), bacterial enzymes (such as hyaluronidase, lipase, phospholipase, and phosphatase), and temperature difference in the infected microenvironment can all be used as stimulus response factors, providing a design basis for the development of intelligent nanomaterials with stimuli responsiveness, of which the intelligent design of pH responsiveness is the most common.^[^ ^222^ ^]^ Under the trigger of acidic pH, the acid‐sensitive chemical bonds of pH‐responsive nanomaterials break, leading to nanomaterial disintegration and encapsulated drug release.^[^ ^225^ ^]^ This intelligent design can avoid premature drug release and reduce toxic effects on normal tissues by distinguishing between the normal pH of physiological environment and the low pH of the infectious microenvironment.^[^ ^226^ ^]^ Previous study has reported methods for fabricating pH‐responsive nanomaterials by using pH‐responsive linkers to connect QSIs to the polysaccharide backbone of alginate NPs, and using charge interactions between the positively‐charged drug and the carboxyl residues of the alginate matrix to encapsulate ciprofloxacin (CIP). When the nanomaterials reach the low‐pH biofilm, the pH‐responsive linker between the QSI and the polysaccharide backbone is cleaved, and the QSI is released. Moreover, due to the carboxyl side chain protonation under acidic conditions, the charge interaction between CIP and alginate matrix is weakened, and CIP is released. Under the dual effects of CIP and the QSI, biofilms are effectively cleared.^[^ ^60^ ^]^ Notably, the efficacy of stimulus‐responsive nanomaterials is limited by the abundance of stimulus response factors. Designing multi‐stimulus responsive nanomaterials with multiple response mechanisms (such as pH/enzyme, pH/temperature, and other dual‐stimuli‐responsive nanocomposites) will effectively overcome this deficiency.

Designing intelligent nanomaterials with controllable sustained release can optimize drug release behaviors in different application scenarios, such as improving the drug concentration or optimizing the release time. This effectively avoids toxic side effects caused by the burst release of the drug and reduces medication frequency while enhancing patient adherence.^[^ ^135^, ^207^ ^]^ The release of nanomaterials is influenced by many factors, such as the interaction between drug molecules and solvent or matrix, drug diffusion in the matrix, and the degradation behavior of the matrix.^[^ ^185^, ^227^ ^]^ The key to achieving controllable sustained release is in the selection of the matrix (including mesoporous silica, lipids, polymers, and metals), among which the lipid matrix is most commonly used for the preparation of controllable sustained‐release nanomaterials with QS regulatory activity.^[^ ^72^, ^206^ ^]^ It has been shown that the poor controlled release ability of traditional solid lipid NPs (SLN) may lead to drug leakage during storage.^[^ ^228^ ^]^ Adding natural QSIs to the lipid matrix of SLN can overcome the shortcomings of traditional SLN and achieve better drug release control by chemically interacting with other pharmaceutical ingredients via hydrogen bonding, halogen interactions, and other bonding types.^[^ ^33^, ^64^, ^206^ ^]^ The metal matrix is also widely used. This type of nanomaterial is mainly prepared by doping another metal with QS regulatory activity in the inert metal matrix. A study has reported the preparation of AgCl‐TiO_2_ NPs (ATNPs) by doping Ag into TiO_2_ matrix. ATNPs have a longer and more effective anti‐QS activity than Ag nanowires, mainly due to the good dispersion and trapping effects of TiO_2_ matrix, which enables long‐term controlled Ag release.^[^ ^35^ ^]^ In addition to the abovementioned matrices, the hydrogel system has advantages such as slow release, low toxicity, biodegradability, and biocompatibility. It is also the focus of attention when designing controllable sustained‐release nanomaterials.^[^ ^229^ ^]^

**Figure 11:**
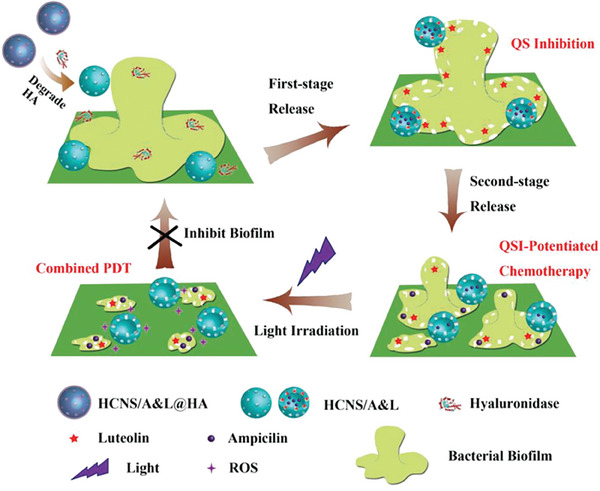
Schematic representation of dispersing biofilm by the bacterial enzyme‐triggered nanosystem, which could modulate QS of bacteria, potentiate antibiotic efficiency, and synergistic photodynamic treatment. Reproduced with permission.^[^ ^231^ ^]^ Copyright 2019, Wiley.

Interestingly, by adjusting the release response mechanism and release rate of the drug combination under different stimuli, designing the intelligent nanosystem with programmed sequential drug release that can control the treatment sequence and duration of the drug combination will improve the efficacy and specificity of the combination.^[^ ^230^ ^]^ As an example, HCNS/A&L@HA is a type of nanomaterials that has been prepared by encapsulating QSI luteolin and antibiotic ampicillin in hollow carbon nitride spheres (HCNSs) capped with hyaluronic acid. QSIs can increase the sensitivity of biofilms to antibiotics; hence, the release sequence of luteolin followed by ampicillin results in a biofilm inhibition rate of up to 64.2% (**Figure** **11**).^[^ ^231^ ^]^

#### 4.1.5 Others

**Figure 12:**
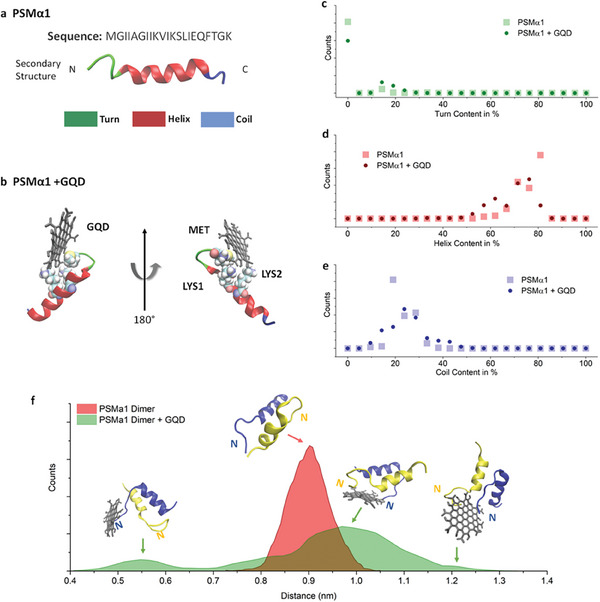
Molecular dynamics simulation of PSMα1 and GQDs. Schemes of a) PSMα1 and b) PSMα1 and GQD complex. β‐Turns are shown in green, α helix in red, and random coils in blue. c–e) Histogram of secondary structure amounts in PSMα1 in the GQD/PSMα1 complex. f) Histogram of the center of mass distance between two PSMα1 units showing the distance distribution with and without a GQD molecule. N: N‐terminal of PSMα1; C: C‐terminal of PSMα1. Reproduced with permission.^[^ ^232^ ^]^ Copyright 2019, American Chemical Society.

Apart from the abovementioned optimization strategies, enhancing the anti‐efflux capacity of nanomaterials is an important strategy for optimizing their QQ activity. Some nanomaterials can be used to overcome the shortcomings of traditional antibiofilm agents that are easily degraded by bacteria and quickly discarded from the biofilm by interacting with biomolecules within the biofilm. Phenol‐soluble modulin (PSM) is a key structural component of the extracellular matrix in biofilms. Graphene Quantum Dot (GQD) docks at the N‐terminus of PSM via its carboxyl group and forms stable conformational complexes (PSM‐GQD), which inhibits bacterial QS while resisting proteolytic enzyme degradation and delaying diffusion and expulsion from biofilms (**Figure** **12**).^[^ ^232^, ^233^ ^]^

### 4.2 Optimization of Environments

**Figure 13:**
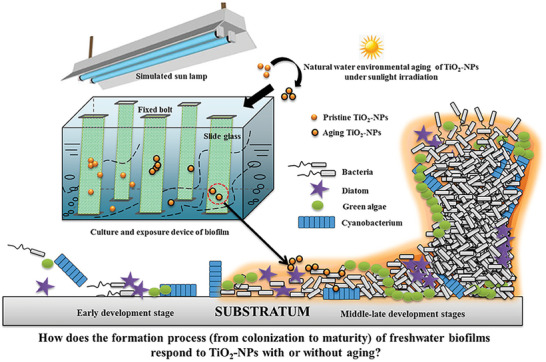
Environment factors cause nanomaterials aging, which affects nanomaterial QS regulatory activity and freshwater biofilm formation processes (from colonization to maturity). Reproduced with permission.^[^ ^175^ ^]^ Copyright 2020, Elsevier.

Besides the properties of the nanomaterial itself, the environment is an important factor that affects nanomaterial QS regulatory activity (**Figure** **13**). The QS regulatory activity of nanomaterials can be improved by optimizing environmental factors (including temperature, light condition, solution environment, and biological factors).^[^ ^14^, ^234^ ^]^

Light can enhance the QS regulatory activity of photocatalytic nanomaterials and photothermal nanomaterials.^[^ ^28^ ^]^ However, illumination is not a necessary condition for these nanomaterials to perform the function of QS regulation, probably because a high dose of nanomaterials can trigger QS regulation through other inefficient mechanisms.^[^ ^14^ ^]^ The type and concentration of ROS whose production is catalyzed by light sources with different wavelengths are also different, which may somewhat affect nanomaterial QS regulatory activity.^[^ ^235^ ^]^ It is worth noting that the QS regulatory activity of these nanomaterials is dose‐dependent; however, this dependence should be within a certain range, because excessive nanomaterials will affect the photoexcitation efficiency and photothermal conversion efficiency.^[^ ^14^ ^]^ Future research should explore the best matching dose of nanomaterials and light intensity to optimize the QS regulatory activity of these nanomaterials.

The QS regulatory activity of nanomaterials is also affected by ambient temperature.^[^ ^110^ ^]^ Increasing ambient temperature can enhance the QS regulatory activity of nanomaterials by promoting metal ion dissolution and ROS production, thereby weakening bacterial tolerance. First, temperature increase can accelerate the transfer of dissolved oxygen and reduce the activation energy required for the reaction, which accelerates the dissolution rate of metal‐based nanomaterials.^[^ ^210^ ^]^ Second, under high temperature conditions, electrons are easily captured at the active sites of metal‐based nanomaterials, and dissolved oxygen can transfer mass to the surface active sites more quickly; then, the interaction between electrons and oxygen produces more ROS.^[^ ^236^ ^]^ Finally, temperature is an important factor affecting bacterial activity. High temperatures can promote nanomaterials absorption by increasing the permeability of bacterial cell membranes, thereby increasing bacterial sensitivity to nanomaterials.^[^ ^237^ ^]^ In the future, the use of photothermal agents will be a promising optimization method to enhance the QS regulatory activity of nanomaterials.

The QS regulatory activity of nanomaterials is also closely related to the pH, organic matter content, ionic strength, and other factors related to the solution environment. A decrease in pH increases the dissolution rate of metal‐based nanomaterials, resulting in stronger QS regulatory activity.^[^ ^238^ ^]^ Under acidic conditions, the protonation of amino groups or the breaking of acid‐sensitive chemical bonds results in cation formation; this can transform the negative or neutral surface of the nanomaterial to a positive surface, which interacts with the negatively charged bacterial surface, thereby increasing the adhesion of the nanomaterial to the bacterial surface.^[^ ^239^ ^]^ Furthermore, compared with the modified Tris minimal media, the nutrient‐rich media increases the concentration of nanomaterials required to inhibit bacterial QS—probably due to the large amount of organic matter adsorbed on the nanomaterial surface in the solution—thereby weakening the adhesion of nanomaterials to bacteria. Moreover, nutrient‐rich media can nourish bacteria and improve their tolerance to nanomaterials.^[^ ^35^ ^]^ The ionic strength in solution can also regulate the QS regulatory activity of nanomaterials by affecting their dissolution equilibrium.^[^ ^240^ ^]^

Biological factors (mainly bacteria) also considerably affect nanomaterial QS regulatory activity.^[^ ^241^ ^]^ The nanomaterial QS regulatory activity is highly dependent on the type of bacteria, probably due to the structural differences of bacterial species and the different bacterial response mechanisms to environmental stress.^[^ ^241^, ^242^ ^]^ Nanomaterials used for specific pathogens can be designed to reduce the occurrence of drug resistance and dysbacteriosis. Bacteria can also weaken the QS regulatory activity of nanomaterials by altering their physicochemical properties.^[^ ^175^ ^]^ For example, bacterial biofilms can change the shape of NPs from spherical to rod‐shaped structures, thereby weakening the toxic effect of NPs on bacteria.^[^ ^10^ ^]^

## 5 Limitations and Prospects

In summary, the use of nanomaterials to regulate bacterial QS is a promising anti‐virulence strategy. This review systematically summarizes the specific regulatory mechanisms and related influencing factors of nanomaterials at different steps of QS, deepening the understanding of how nanomaterials regulate bacterial communication and group behaviors. More importantly, based on the two key influencing factors of “subject and environment” (i.e., the nanomaterial itself and the environment), the specific optimization strategies to enhance the QS regulatory activity of nanomaterials are comprehensively summarized. It is expected that this review will serve as a reference for the rational design and development of novel, intelligent antibacterial nanomaterials with higher efficiency and more comprehensive functions in the future. However, there are still some limitations in current research, which hinder the future development prospects of this strategy. A better understanding of the potential development constraints and an exploration of possible improvement directions will enhance the application potential of the strategy.

### 5.1 Breadth‐Depth Dilemma

In terms of research depth, most existing studies have focused on in vitro analyses; in contrast, relatively few studies report in‐vivo analyses, probably because the endpoint effect of QSIs is bacterial virulence inhibition rather than direct bacterial killing, making it difficult to reflect the QS regulatory activity of nanomaterials solely through typical in‐vivo research endpoints such as bacterial load.^[^ ^26^ ^]^ Furthermore, constructing an intuitive in vivo research model constitutes a challenge in conducting certain in vivo QS studies.^[^ ^243^ ^]^ Considering that in‐vivo research is an essential procedure before the clinical application of nanomaterial with QS regulatory activity, it is necessary to refine the relevant in vivo studies with respect to the following aspects in the future: the actual bioavailability of nanomaterials with QS regulatory activity in complex internal environments (such as biochemical barrier, mucus barrier and cell barrier), the toxicological and pharmacokinetic study of nanomaterial with QS regulatory activity in the host, and the effect of nanomaterial with broad‐spectrum QS regulatory activity on host microbial homeostasis and immune regulation.^[^ ^244^ ^]^ Besides the lack of in‐vivo studies, most studies have only confirmed at the phenomenological level that nanomaterials have QS regulatory effects (such as reducing the production of QS‐regulated virulence factors, inhibiting swarming and swimming motility, and disrupting biofilm formation), while research on the specific molecular mechanisms by which nanomaterials regulate bacterial QS is relatively limited.^[^ ^245^ ^]^ The possible reason is that existing mechanistic research mostly relies on computerized molecular docking techniques to simply predict whether there is a direct interaction between nanomaterials and known QS system components.^[^ ^150^, ^246^ ^]^ The introduction of more advanced analytical tools in the future, such as high‐resolution single‐atom imaging techniques, is expected to break through this limitation.^[^ ^234^ ^]^ Meanwhile, more in‐depth research on the following related mechanisms should be considered: (1) a further exploration of the specific role of bacterial metabolic pathways in the QS regulatory mechanisms of nanomaterials. Whole genome RNA sequencing and quantitative proteomic analysis revealed significant changes in bacterial metabolic pathways (including iron homeostasis, redox balance, amino acid metabolism, and respiration) after exposure to nanomaterials with QS regulatory activity.^[^ ^13^, ^56^, ^81^, ^99^ ^]^ In the future, further clarification of the role of the abovementioned metabolic pathways in bacterial QS regulation by nanomaterials will enable in‐depth exploration of the mechanisms. (2) evaluation of the influence of nanomaterials with QS regulatory activity on the signal transmission network related to virulence generation. QS and several other regulatory mechanisms (involving regulatory RNA, second messengers, and sigma factor) are interconnected to form a complex signal transmission network and jointly participate in controlling the production of virulence factors of pathogenic bacteria.^[^ ^171^ ^]^ In the future, clarifying the mechanism of action of nanomaterials on this signal transmission network will help to design and develop new multi‐target nanomaterials with better antibacterial effects.

In terms of research breadth, first of all, current research is mostly limited to the impact of nanomaterials on the QS of single bacterial strain; nevertheless, little attention is paid to the QS between different strains, probably because of the following reasons: (1) the presence of many confounding factors in multi‐strain research, making it difficult to control variables for research and analysis; and (2) a lack of multi‐strain standard model. Despite attempts to introduce in vitro 2D models, such as polymicrobial biofilm keratinocyte colonization model and microfluidic devices, these models still differ significantly from the complex in vivo environment.^[^ ^105^, ^247^ ^]^ And the organoid models derived from human progenitor cells may be an effective way to make up for the differences between models in vitro and in vivo.^[^ ^248^ ^]^ Furthermore, most of the current studies apply nanomaterials with QS regulatory activity before the establishment of infection to evaluate their role in infection prevention. However, there is still a lack of related studies evaluating the therapeutic potential of nanomaterials with QS regulatory activity in infection control. Finally, fungi also have complex QS systems. The regulatory effect of nanomaterials on communication in fungi and multi‐species mixed biofilms is also an interesting research field.^[^ ^249^ ^]^

### 5.2 Quorum Quenching Resistance

Existing studies have confirmed that bacteria have resistance to the QQ activity of nanomaterials, but the related research is still limited.^[^ ^144^ ^]^ Current research has mostly confirmed at the phenotypic level that bacteria are resistant to the QQ effect of nanomaterials, which is related to the adaptive evolution of bacteria. However, there is little understanding of the underlying molecular mechanisms. Recent studies have found that some metal‐based nanomaterials can interact with the binding pocket of the LasR protein to directly activate QS gene expression related to bacterial biofilm formation, thereby promoting bacterial adaptive evolution.^[^ ^144^ ^]^ In the future, it is essential to gain a better understanding of the molecular mechanisms underlying nanomaterial QQ resistance. This understanding will help us develop strategies for the application of nanomaterials to control specific pathogenic bacteria while simultaneously avoiding the risk of QQ resistance. In addition to the current lack of understanding of the underlying molecular mechanisms, current studies on the resistance of bacteria to nanomaterial QQ activity focus on resistance and tolerance; less attention is paid to the important role of persistence. However, recent studies have found that nanoalumina can trigger antibiotic persistence of *E. coli* through the QS factors *lrsF* and *qseB*.^[^ ^250^ ^]^ Persistent bacteria can avoid the bactericidal effect of antibiotics by maintaining a dormant state with no metabolic activity. Once the drug pressure is eliminated, the bacteria quickly return to the wild type, resulting in recalcitrant infections. Therefore, future studies should closely consider the role of persistence in the resistance of nanomaterial QQ activities.^[^ ^251^ ^]^

Most experts believe that this QQ resistance will seriously weaken the anti‐virulence effect of nanomaterials. Future studies should provide solutions to the problem of nanomaterial QQ resistance, which is a key issue in the practical application of nanomaterials to regulate bacterial QS. To address this issue, the following aspects can be considered in future research: (1) reverse bacterial resistance by adjusting medication administration strategies to re‐sensitize bacteria to nanomaterials. At present, this strategy is mostly used for persistent bacteria, such as the application of pulse dosing strategy, which interrupts treatment by pausing medication to promote bacterial exit from the dormant state and re‐sensitize cells to drugs.^[^ ^252^ ^]^ (2) weaken the ability of resistant bacteria to resist drugs by developing nanomaterials targeting the core components of QS networks as well as higher‐level QS systems or by selecting appropriate drug combinations. This strategy is mainly aimed at situations where inhibiting a single QS system may lead to the resistance or compensatory activation of parallel QS systems.^[^ ^8^ ^]^ (3) develop a more effective and targeted novel nanomaterial with anti‐QS activity based on the specific molecular mechanism of QQ resistance, thereby reducing toxic side effects while retarding the occurrence of QQ resistance of non‐target bacterial strains to the greatest extent.^[^ ^253^ ^]^ (4) clarify the optimal dosage of nanomaterials for regulating bacterial QS to avoid the occurrence of QQ resistance induced by inappropriate drug dosage.^[^ ^103^, ^253^ ^]^

However, some experts believe QQ resistance should not be a point of concern because some mutator strains need to rely on the QS‐dependent products of non‐mutator strains to maintain survival. When the QS activity of non‐mutator strains is quenched, the mutator strains cannot survive due to the lack of sufficient QS‐dependent products. Furthermore, QS may be more beneficial for the survival of certain bacteria in complex environments. Mutator strains may not strongly adapt to the environment compared with non‐mutator strains due to a lack of complete QS function.^[^ ^108^, ^254^ ^]^

### 5.3 Others

Currently, research has mainly focused on the unidirectional regulatory effect of nanomaterials on bacterial QS, with less attention paid to the “counteraction” of bacterial QS on nanomaterials. However, research has found that this “counteraction” may decrease the stability of nanomaterials, thereby weakening their original QS regulatory activity. For example, Au NPs are widely used to stabilize QQ enzymes and regulate bacterial QS due to their extremely stable physicochemical properties.^[^ ^255^ ^]^ Nevertheless, some bacteria can regulate the production of cyanide through QS, resulting in a rapid oxidative dissolution of Au NP and an inability to exert its original QS regulatory function.^[^ ^256^ ^]^ Similarly, studies have found that certain bacterial biofilms can convert spherical nanomaterials with stronger QS regulatory activity into rod‐shaped structures with weaker regulatory activity, although the detailed mechanisms are unclear.^[^ ^10^ ^]^ Future studies should evaluate the “counteraction” of bacterial QS on nanomaterials to comprehensively evaluate the practical application effects of nanomaterials with QS regulatory activity.

In some cases, the anti‐virulence strategies using nanomaterials to quench bacterial QS may increase the prevalence of more virulent genotypes within the host or indirectly exert selective pressure on antibiotic resistance.^[^ ^257^ ^]^ Therefore, before the practical clinical application of nanomaterials with QS regulatory activity, more in vivo and preclinical data are needed to evaluate the potential drug resistance risk of nanomaterials entering the human body.

## Conflict of Interest

The authors declare no conflict of interest.
